# Supplementary material for: Use of artificial intelligence in the detection of primary prostate cancer in multiparametric MRI with its clinical outcomes: a protocol for a systematic review and meta-analysis
Source: BMJ Open. 2023 Aug 22;13(8):e074009. doi: 10.1136/bmjopen-2023-074009 (PMC10445392; doi:10.1136/bmjopen-2023-074009)
Supplement: Supplementary data [file bmjopen-2023-074009supp001.pdf]

The use of artificial intelligence in the detection of primary prostate cancer in multiparametric magnetic resonance imaging with its clinical outcomes: a protocol for a systematic review and meta-analysis

The search terms for this systematic review are as follows:

((artificial intelligen* OR machine intelligen* OR computational intelligen* OR neural network OR deep learning OR machine learning OR reinforcement learning OR convolutional neural network OR artificial neural network OR recurrent neural network OR deep neural network OR intelligent model OR AI OR NN OR DL OR ML OR CNN OR ANN OR RNN OR DNN) AND ((Prostat* AND (cancer OR malignan* OR adenocarcinoma OR lesion* OR disease)) AND (MR OR magnetic resonance imaging OR MP-MRI OR multi-parametric MRI OR multi-parametric magnetic resonance imaging OR multiparametric MRI OR “multiparametric magnetic resonance imaging” OR radiomic* OR radiogenomic*)) AND diagnosis).ti,ab

Searches will be conducted on the following databases:

PubMed, MEDLINE, Embase, and Cochrane databases
