# Supplementary material for: Use of artificial intelligence in the detection of primary prostate cancer in multiparametric MRI with its clinical outcomes: a protocol for a systematic review and meta-analysis
Source: BMJ Open. 2023 Aug 22;13(8):e074009. doi: 10.1136/bmjopen-2023-074009 (PMC10445392; doi:10.1136/bmjopen-2023-074009)
Supplement: Author's manuscript [file bmjopen-2023-074009.draft_revisions.pdf]

BMJ Open is committed to open peer review. As part of this commitment we make the peer review history of every article we publish publicly available.

When an article is published we post the peer reviewers' comments and the authors' responses online. We also post the versions of the paper that were used during peer review. These are the versions that the peer review comments apply to.

The versions of the paper that follow are the versions that were submitted during the peer review process. They are not the versions of record or the final published versions. They should not be cited or distributed as the published version of this manuscript.

BMJ Open is an open access journal and the full, final, typeset and author-corrected version of record of the manuscript is available on our site with no access controls, subscription charges or pay-per-view fees (http://bmjopen.bmj.com).

If you have any questions on BMJ Open's open peer review process please email info.bmjopen@bmj.com

BMJ Open

The use of artificial intelligence in the detection of primary prostate cancer in multi-parametric magnetic resonance imaging with its clinical outcomes: a protocol for a systematic review and meta-analysis

Journal:	*BMJ Open*
Manuscript ID	bmjopen-2023-074009
Article Type:	Protocol
Date Submitted by the Author:	24-Mar-2023
Complete List of Authors:	Thomas, Maya; Imperial College London, School of Medicine<br>Murali, Sanjana; Imperial College London, School of Medicine<br>Simpson, Benjamin Scott; University College London, UCL Cancer Institute<br>Freeman, Alex; University College London Hospitals NHS Foundation Trust, Department of Pathology<br>Kirkham, Alex; University College London Hospitals NHS Foundation Trust, Department of Radiology<br>Kelly, Daniel; Cardiff University, School of Healthcare Sciences<br>Whitaker, Hayley; University College London, UCL Division of Surgery and Interventional Science<br>Zhao, Yi; Imperial College London, School of Medicine<br>Emberton, Mark; University College London, UCL Division of Surgery & Interventional Science; University College London Hospitals NHS Foundation Trust, Department of Urology<br>Norris, Joseph; University College London, UCL Division of Surgery & Interventional Science; University College London Hospitals NHS Foundation Trust, Department of Urology
Keywords:	Prostate, Urological tumours < UROLOGY, Urological tumours < ONCOLOGY, RADIOLOGY & IMAGING, Magnetic resonance imaging < RADIOLOGY & IMAGING

SCHOLARONE™
Manuscripts

The use of artificial intelligence in the detection of primary prostate cancer in multi-parametric magnetic resonance imaging with its clinical outcomes: a protocol for a systematic review and meta-analysis

Maya Thomas[1], Sanjana Murali[1], Benjamin S. Simpson[2], Alex Freeman[3], Alex Kirkham[4], Daniel Kelly[5], Hayley C. Whitaker[6], Yi Zhao[1], Mark Emberton[6,7], Joseph M. Norris[6,7]

MT and SM are joint first authors.

ME and JMN are joint senior authors.

[1] Imperial College London School of Medicine, London, UK

[2] UCL Cancer Institute, University College London, London, UK

[3] Department of Pathology, University College London Hospitals NHS Foundation Trust, London, UK

[4] Department of Radiology, University College London Hospitals NHS Foundation Trust, London, UK

[5] School of Healthcare Sciences, Cardiff University, Wales, UK

[6] UCL Division of Surgery & Interventional Science, University College London, London, UK

[7] Department of Urology, University College London Hospitals NHS Foundation Trust, London, UK

Correspondence to: yi.zhao18@imperial.ac.uk

For peer review only

Imperial College London

Exhibition Rd, South Kensington, London SW7 2BU

yi.zhao18@imperial.ac.uk

Ethics: No ethical considerations apply.

Word count: 1864

For peer review only

ABSTRACT

Introduction: Multiparametric magnetic resonance imaging (mpMRI) has transformed the prostate cancer diagnostic pathway, allowing for improved risk stratification and more targeted subsequent management. However, there exist concerns over the interobserver variability of images and the applicability of this model long term, especially considering the current shortage of radiologists and the growing ageing population. Artificial intelligence (AI) is being integrated into clinical practice to support diagnostic and therapeutic imaging analysis to overcome these concerns. The following report details a protocol for a systematic review and meta-analysis investigating the accuracy of AI in predicting primary prostate cancer on mpMRI.

Methods and Analysis: A systematic search will be performed using PubMed, MEDLINE, Embase and Cochrane databases. All relevant articles published between July 1977 and February 2023 will be eligible for inclusion. To be included, articles must use AI to study MRI prostate images to detect prostate cancer. All included articles will be in full-text reporting original data and written in the English language. The Preferred Reporting Items for Systematic Review and Meta-Analysis (PRISMA) guidelines will direct the screening, data extraction, statistical analysis and reporting process. The QUADAS-2 score will be employed to assess the quality and risk of bias across selected studies. This protocol is written in line with the PRISMA-protocols 2015 checklist.

Ethics and dissemination: Ethical approval will not be required for this systematic review. Findings will be disseminated through peer-reviewed publications and presentations at both national and international conferences.

PROSPERO registration number: CRD42021293745

For peer review only

Strengths and limitations of this study:

- This study will be the first systematic review and meta-analysis to look solely at AI algorithms for mpMRI and see how they compare to expert evaluation, written in line with the PRISMA guidelines
- This study will utilise comprehensive statistical analysis to try and mitigate the effects of heterogenous reporting methods and varied endpoints in the determined results
- The strength of the conclusions may be limited by the abilities of the trained AI in selected studies, affected by the amount and variety of data the original AI algorithms were trained using
- As AI is a relatively novel technology in mpMRI, the long-term outcomes associated with these studies may be limited

For peer review only

BACKGROUND

Accurately identifying clinically significant prostate cancer has been a long-standing, challenging issue. Previous diagnostic pathways have led many patients undergoing biopsies to be diagnosed with clinically insignificant prostate cancer. This can lead to potentially aggressive overtreatment, unnecessary healthcare cost and detrimental effects on patient mental health[1-3].

The introduction of multiparametric magnetic resonance imaging (mpMRI) was a pivotal event in resolving this issue. Multiple sources now recommend using mpMRI as a prebiopsy investigation[2-7] to allow earlier risk stratification of those with suspected prostate cancer and improve the utility of the biopsies performed by enabling a more targeted approach. Despite this, interobserver variability of images remains a major concern[5,8], with a lack of standardised protocols and a requirement for experienced radiologists being examples of contributory factors [4,9]. While radiology trainees show rapid

For peer review only

improvement in the accuracy of interpreting studies with increased exposure, there is still a margin of error at around 25%[10]. Additionally, prostate cancer is the second most frequent cancer diagnosis globally and the healthcare burden is only set to increase due to a growing ageing population, therefore the volume of diagnostic imaging and management monitoring required of radiologists will also likely increase over time[11].

Artificial Intelligence (AI) including Deep Learning (DL) describes how computers can be programmed to imitate human intelligence[12]. Neural networks are a subset of deep learning models that mimic the human visual cortex[13]. Each layer of a neural network comprises neurons that identify several features of an image by applying multiple edge, colour and texture filters[14]. These neural networks are trained to associate specific characteristics with certain classifications by iterating through a training dataset of images. These trained layers can work together to perform feature identification on unseen photos; for example, in mpMRI, they could segment a potentially suspicious lesion in the prostate[15,16]. AI is being integrated into clinical practice to support diagnoses, assist in therapeutic decisions, and predict patient outcomes[17]. Several studies have assessed the level of agreement between AI and DL to clinical radiologists to interpret mpMRI in prostate cancer investigation[18-21]. Application of AI could improve the current interobserver variability, expedite mpMRI interpretation[20], and risk-stratify potentially suspicious lesions for radiologist review.

However, it is also essential to address that DL algorithms are not yet advanced enough for unsupervised clinical utilisation[18,22]. Many issues surround the training of AI models, such as lack of diversity in training datasets – primarily in ethnicity, with many cohorts using predominantly white

For peer review only

patients[23], even though prostate cancer is more prevalent and aggressive in Afro-Caribbean men[11]. In many studies, there is a lack of external validation, which makes it unclear how models would perform on new data, and also runs the risk of overfitting to training data – where the model is excellent at recognising lesions in training data but is unable to recognise lesions in new data[23]. Class imbalance due to the majority of negative cases can also present as an issue by creating algorithm bias and false negatives, which warrants review by senior radiologists[23].

Furthermore, variations of the Prostate Imaging Reporting and Data System (PI-RADS) exist– with version 1 and version 2 in use [24]. This makes evaluating how comparable the AI is more challenging due to a lack of consistency. This study will explore this in more detail to inform future reporting methodologies.

This systematic review and meta-analysis aim to summarise the accuracy of AI in predicting prostate cancer on mpMRI and how its use may impact clinical outcomes in patients.

METHODS AND ANALYSIS

The protocol for this systematic review is written in line with the Preferred Reporting Items for Systematic Review and Meta-Analysis Protocols (PRISMA-P) 2015 checklist[25]. The study has been prospectively

For peer review only

registered with PROSPERO review databases (CRD42021293745), and all methods described here were established before implementation. The statistical data evaluating the sensitivity, specificity, positive predictive value (PPV), negative predictive value (NPV), and area-under-the-curve (AUC) of the use of AI in mpMRI prostate imaging for the detection of prostate cancer will be derived following a thorough analysis and thematic synthesis of included studies. These studies' pooled sensitivities and specificities will be determined before PPV and NPV values are derived.

Search Methodology

A systematic search will be performed using PubMed, MEDLINE, Embase and Cochrane databases. The search strategy will include using medical subject heading (MeSH) terms and free text with appropriate Boolean operators and will encompass articles from July 1977 (first human MRI) to February 2023 to achieve maximum yield of the relevant evidence. The search will include the following key terms: "prostate", "cancer", "diagnosis", and multiple synonyms for "mpMRI", "artificial intelligence", and "deep learning". To facilitate the initial screening process, Rayyan will be employed, a semiautomated application designed to improve the speed and reporting accuracy of systematic reviews[26]. All eligible articles from the initial search will be uploaded to Rayyan. A further manual search of the references in all included articles will be performed to identify any further relevant literature not found by the initial search strategy. If data is absent or ambiguous, the corresponding authors will be contacted for clarification.

For peer review only

Study selection and data extraction

The screening process will be completed independently by two researchers (M.T. and S.M.). Titles and abstracts of eligible studies will be assessed, and irrelevant articles will be removed. A full-text version of relevant articles will be downloaded for further eligibility review. Any dispute amongst researchers will be discussed and resolved by consensus, or a third reviewer (Y.Z.) will be consulted. The reasoning for excluding articles will be documented and detailed in a PRISMA flow diagram. Before commencing the screening process, calibration exercises will be conducted to maintain consistency between the researchers, reducing inter-reviewer bias.

Inclusion and exclusion criteria

For inclusion in this systematic review, studies must use AI to study MRI prostate images to detect prostate cancer. Prospective or retrospective studies can be included. Comparisons will be focussed on evaluating sensitivities, specificities, PPVs, NPVs and AUCs.

Correspondence papers, ongoing studies, case reports and conference abstracts reports will be excluded from this analysis. Articles which are not written in the English Language will also be excluded.

Data extraction (table of collection)

For peer review only

170 The following data in table 1 will be collected from all included studies. The researchers will perform data extraction independently, with collected data collated in a dedicated datasheet. Any discrepancies in data extraction will be resolved by consensus.

Table 1. Data collection items

ITEM NO.	DATA TITLE	DATA TYPE
1	Year of publication	Study characteristic
2	Study authors	Study characteristic
3	Experimental design	Study characteristic
4	Patient population	Demographics
5	Study size	Demographics
6	Type of MRI imaging used	Methodology
7	Type of AI models used	Methodology
8	Definition of clinically significant disease	Methodology
9	Predictive performances	Outcome

AI: artificial intelligence; MRI: magnetic resonance imaging

Endpoints

The primary endpoint will be statistically significant quantified accuracy in the use of AI in MRI prostate imaging to detect prostate cancer and determine whether AI could be deployed in the clinical decision-

For peer review only

making process. Additional outcomes will include other parameters investigating the extent of the disease.

Meta-analysis

A meta-analysis will be conducted if there are a sufficient number of appropriate studies. This would aim to obtain a pooled quantitative diagnostic accuracy value for AI performance in detecting prostate cancer from mpMRI. To begin, sensitivity and specificity values will be retrieved, or if studies do not provide these values, they will be calculated from clinical tables or requested from corresponding authors. If a considerable proportion of included studies used another metric, these values would be retrieved and calculated separately. The distributions of the untransformed, logit and double-arcsine transformed proportions will be compared and assessed for normality using density plots and Shapiro-Wilk tests, with whichever set of ratios most resembling normal distribution being used for further analysis.

Inter-study variation will be quantified through I^2 and if statistically significant, a random-effects model will be fitted for estimation of the summary estimate. Once the model fits all relevant studies, leave-one-out analysis (LOO) and accompanying diagnostic plots will be used to identify influential studies. This will include externally studentised residuals, the difference in fits values (DFFITS), Cook's distances, covariance ratios, LOO estimates of the amount of heterogeneity, LOO values of the test statistics for heterogeneity, hat values and weights. Each study will be removed one at a time, and the summary

For peer review only

proportions re-estimated based on the remaining n-1 studies. Studies with a statistically significant influence on the fitted model will be considered outliers and removed. The model will then be re-fitted. A summary estimate comprising the remaining studies will then be calculated to estimate the accuracy using AI in mpMRI for prostate cancer detection. All data analysis and visualisation will be performed using the R statistical environment using the “mvmeta” and “meta” packages.

Risk of bias in individual studies

The QUADAS-2 score will be employed to assess the quality and the risk of bias across the selected studies[27]. This tool comprises four domains: patient selection, index test, reference standard, and flow and timing. The quality of the research methodology for each study will be assessed within these four domains. Two (M.T. and Y.Z.) reviewers will be independently involved in this process, with any disparities resolved by consensus. The bias assessment will evaluate the applicability and reliability of the data used. This will 7-8inform evidence synthesis and increase transparency as articles may be excluded if found to be of low quality or suggestive of high levels of bias, or if included, appropriate commentaries will be incorporated into the discussion.

Patient and public involvement

There will be no patient or public involvement in this study.

DISCUSSION

For peer review only

As a primary imaging modality, mpMRI has many uses, established in pre-biopsy risk stratification, active surveillance, and treatment planning[3]. AI and DL have substantial roles in streamlining service provision and optimising workloads for radiologists, however, the potential role of AI in assisting mpMRI interpretation for prostate cancer diagnosis is yet to be evaluated. We intend to review the performance of existing AI algorithms in mpMRI diagnosis of primary prostate cancer. Through the summation of this information, we hope to clarify the role of AI in improving reporting and summarise its overall impact on clinical care.

To date, most of the relevant evidence compares AI reporting alone to radiology reporting. Some of these studies report promising results – that AI is generally accurate and has the potential to be incorporated more permanently as an evaluation and reporting tool[19]. However, the accuracy of AI reporting tends to depend on the variety of the training data set and the variety of the internal and external validation data sets; this has been shown in similar studies concerning radiology reporting in other specialities[28]. This review will evaluate AI reporting of mpMRI images and will comment on the variety of datasets used in current evidence, therefore encouraging further research in this area.

In terms of potential limitations, we recognise that due to its relative novelty, there may be a limited number of studies that have evaluated AI in this context. There could be issues surrounding the heterogeneity of reporting protocols due to local variation. Different neural networks may also act as confounders due to different training datasets, which would arguably report differently depending on learned features.

For peer review only

Overall, this systematic review and meta-analysis will combine the existing literature and allow for the much-needed evaluation of AI and DL for mpMRI in diagnostic processes. The synthesis of this data will result in a better understanding of current AI and DL protocols and how existing systems can be further improved for supervised and unsupervised usage. We envisage this process will highlight areas requiring further research and will help optimise reporting protocols in the future, therefore aiding prostate cancer diagnosis and improving patient outcomes.

Trial status:

- Preliminary searches: Started
- Piloting of the study selection process: Started
- Formal screening: Not started
- Data extraction: Not started
- Risk of bias assessment: Not started
- Data analysis: Not started

Draft search strategy

(Artificial intelligence OR Neural Network OR Deep learning OR AI OR NN OR DL OR CNN OR ANN) AND ((Prostat* AND (Cancer OR malignan* OR adenocarcinoma OR lesion* OR Disease)) AND (MP-

For peer review only

MRI OR multi-parametric MRI OR multi-parametric magnetic resonance imaging OR multiparametric MRI OR "multiparametric magnetic resonance imaging")) AND Diagnosis).ti,ab

Ethics and Dissemination

Due to the nature of this review, there are no relevant ethical concerns and informed consent will not be required. The protocol and systematic review will be disseminated via a peer-reviewed journal.

For peer review only

Author Affiliations

[1] Imperial College London School of Medicine, London, UK

[2] UCL Cancer Institute, University College London, London, UK

[3] Department of Pathology, University College London Hospitals NHS Foundation Trust, London, UK

[4] Department of Radiology, University College London Hospitals NHS Foundation Trust, London, UK

[5] School of Healthcare Sciences, Cardiff University, Wales, UK

[6] UCL Division of Surgery & Interventional Science, University College London, London, UK

[7] Department of Urology, University College London Hospitals NHS Foundation Trust, London, UK

Email: Maya Thomas: maya.thomas18@imperial.ac.uk, Sanjana Murali: sanjana.murali18@imperial.ac.uk, Yi Zhao: yi.zhao18@imperial.ac.uk, Benjamin S. Simpson: b.simpson@ucl.ac.uk, Alex Freeman: alex.freeman2@nhs.net, Alex Kirkam: alexkirkham@nhs.net,

Daniel Kelly: kellydm@cardiff.ac.uk, Hayley C. Whitaker: hayley.whitaker@ucl.ac.uk, Mark Emberton: m.emberton@ucl.ac.uk, Joseph M. Norris: joseph.norris@ucl.ac.uk

Twitter Benjamin S. Simpson @b3nsimp, Daniel Kelly @profdkelly, Hayley C. Whitaker @HayleyWhitaker, Mark Emberton @EmbertonMark, Joseph M. Norris @MrJosephNorris

Contributors The authors' contribution includes, but is not limited to, the following: MT and SM wrote the manuscript. YZ and JMN created the study concept. YZ, BSS, AF, AK, DK, HCW and ME provided supervision and guidance. All authors reviewed and approved the manuscript in its current form. MT and SM are the guarantors of this work.

Funding Norris is funded by the Medical Research Council (MRC) (MR/S00680X/1). Simpson is funded by the Royal Marsden Cancer Charity. Emberton receives research support from the United Kingdom's National Institute of Health Research (NIHR) UCLH/UCL Biochemical Research Centre.

Competing Interests Norris receives funding from the MRC. Simpson receives funding from the Royal Marsden Cancer Charity. Whitaker receives funding from Prostate Cancer UK, the Urology Foundation and Rosetrees Trust. Kirkham, Freeman and Emberton have stock interest in Nuada Medical Ltd. Emberton acts as a consultant, trainer and proctor to Sonatherm Inc; Angiodynamics Inc; Exact Imaging Inc.

Patient consent for publication Not required

For peer review only

Provenance and peer review Not commissioned; externally peer-reviewed

Open access This is an open-access article distributed in accordance with the Creative Commons Attribution 4.0 Unreported (CC BY 4.0) license which permits others to copy, redistribute, remix, transform and build upon this work for any purpose, provided the original work is properly cited, a link to the licence is given, and indication of whether changes were made. See https://creativecommons.org/licenses/by/4.0/.

ORCID IDs

- Sanjana Murali https://orcid.org/0000-0001-7980-6733
- Maya Thomas https://orcid.org/0000-0001-6997-9438
- Yi Zhao https://orcid.org/0000-0002-4563-4344
- Naomi Morka https://orcid.org/0000-0002-1925-7223
- Joseph M. Norris http://orcid.org/0000-0003-2294-0303
- Benjamin S. Simpson http://orcid.org/0000-0003-3685-6110
- Daniel Kelly http://orcid.org/0000-0002-1847-0655
- Hayley C. Whitaker https://orcid.org/0000-0002-2695-0202

References

(1) Sandhu GS, Andriole GL. Overdiagnosis of Prostate Cancer. *Journal of the National Cancer Institute. Monographs.* 2012; 2012 (45): 146-151. 10.1093/jncimonographs/lgs031.

(2) Kasivisvanathan V, Rannikko AS, Borghi M, Panebianco V, Mynderse LA, Vaarala MH, et al. MRI-Targeted or Standard Biopsy for Prostate-Cancer Diagnosis. *New England Journal of Medicine.* 2018; 378 (19): 1767-1777. 10.1056/NEJMoa1801993.

(3) Brown LC, Ahmed HU, Faria R, El-Shater Bosaily A, Gabe R, Kaplan RS, et al. Multiparametric MRI to improve detection of prostate cancer compared with transrectal ultrasound-guided prostate biopsy alone: the PROMIS study. *Health Technology Assessment.* 2018; 22 (39): 1-176. 10.3310/hta22390.

(4) Zhen L, Liu X, Yegang C, Yongjiao Y, Yawei X, Jiaqi K, et al. Accuracy of multiparametric magnetic resonance imaging for diagnosing prostate Cancer: a systematic review and meta-analysis. *BMC cancer.* 2019; 19 (1): 1244. 10.1186/s12885-019-6434-2.

(5) Richenberg J, Logager V, Panebianco V, Rouviere O, Villeirs G, Schoots I. The primacy of multiparametric MRI in men with suspected prostate cancer. *European Radiology.* 2019; 29 (12): 6940-6952. 10.1007/s00330-019-06166-z.

(6) van der Leest M, Cornel E, Israël B, Hendriks R, Padhani AR, Hoogenboom M, et al. Head-to-head Comparison of Transrectal Ultrasound-guided Prostate Biopsy Versus Multiparametric Prostate Resonance Imaging with Subsequent Magnetic Resonance-guided Biopsy in Biopsy-naïve Men with Elevated Prostate-specific Antigen: A Large Prospective Multicenter Clinical Study. *European Urology.* 2019; 75 (4): 570-578. 10.1016/j.eururo.2018.11.023.

(7) Ahmed HU, El-Shater Bosaily A, Brown LC, Gabe R, Kaplan R, Parmar MK, et al. Diagnostic accuracy of multi-parametric MRI and TRUS biopsy in prostate cancer (PROMIS): a paired validating confirmatory study. *Lancet (London, England).* 2017; 389 (10071): 815-822. 10.1016/S0140-6736(16)32401-1.

(8) Stabile A, Giganti F, Rosenkrantz AB, Taneja SS, Villeirs G, Gill IS, et al. Multiparametric MRI for prostate cancer diagnosis: current status and future directions. *Nature Reviews. Urology.* 2020; 17 (1): 41-61. 10.1038/s41585-019-0212-4.

For peer review only

(9) Dominguez C, Plata M, Cataño JG, Palau M, Aguirre D, Narvaez J, et al. Diagnostic accuracy of multiparametric magnetic resonance imaging in detecting extracapsular extension in intermediate and high - risk prostate cancer. *International Brazilian Journal of Urology : official journal of the Brazilian Society of Urology.* 2018; 44 (4): 688-696. 10.1590/S1677-5538.IBJU.2016.0485.

(10) Rosenkrantz AB, Ayoola A, Hoffman D, Khasgiwala A, Prabhu V, Smereka P, et al. The Learning Curve in Prostate MRI Interpretation: Self-Directed Learning Versus Continual Reader Feedback. *AJR. American journal of roentgenology.* 2017; 208 (3): W92-W100. 10.2214/AJR.16.16876.

(11) Rawla P. Epidemiology of Prostate Cancer. *World Journal of Oncology.* 2019; 10 (2): 63-89. 10.14740/wjon1191.

(12) Gore JC. Artificial intelligence in medical imaging. *Magnetic resonance imaging.* 2020; 68 A1-A4. 10.1016/j.mri.2019.12.006.

(13) van Dyck LE, Kwitt R, Denzler SJ, Gruber WR. Comparing Object Recognition in Humans and Deep Convolutional Neural Networks—An Eye Tracking Study. *Frontiers in Neuroscience.* 2021; 15 750639. 10.3389/fnins.2021.750639.

(14) Saha A, Fadaiefard P, Rabski J, Sadeghian A, Cusimano M. *Machine learning applications using diffusion tensor imaging of human brain: A PubMed literature review.* ; 2020.

(15) Kriegeskorte N, Golan T. Neural network models and deep learning. *Current biology: CB.* 2019; 29 (7): R231-R236. 10.1016/j.cub.2019.02.034.

(16) Schelb P, Kohl S, Radtke JP, Wiesenfarth M, Kickingereder P, Bickelhaupt S, et al. Classification of Cancer at Prostate MRI: Deep Learning versus Clinical PI-RADS Assessment. *Radiology.* 2019; 293 (3): 607-617. 10.1148/radiol.2019190938.

(17) Briganti G, Le Moine O. Artificial Intelligence in Medicine: Today and Tomorrow. *Frontiers in Medicine.* 2020; 7 27. 10.3389/fmed.2020.00027.

(18) Syer T, Mehta P, Antonelli M, Mallett S, Atkinson D, Ourselin S, et al. Artificial Intelligence Compared to Radiologists for the Initial Diagnosis of Prostate Cancer on Magnetic Resonance Imaging: A Systematic Review and Recommendations for Future Studies. *Cancers.* 2021; 13 (13): 3318. 10.3390/cancers13133318.

(19) Van Booven DJ, Kuchakulla M, Pai R, Frech FS, Ramasahayam R, Reddy P, et al. A Systematic Review of Artificial Intelligence in Prostate Cancer. *Research and Reports in Urology.* 2021; 13 31-39. 10.2147/RRU.S268596.

For peer review only

(20) Soerensen SJC, Fan RE, Seetharaman A, Chen L, Shao W, Bhattacharya I, et al. Deep Learning Improves Speed and Accuracy of Prostate Gland Segmentations on Magnetic Resonance Imaging for Targeted Biopsy. *The Journal of Urology.* 2021; 206 (3): 604-612. 10.1097/JU.0000000000001783.

(21) Gentile F, La Civita E, Della Ventura B, Ferro M, Cennamo M, Bruzzese D, et al. A Combinatorial Neural Network Analysis Reveals a Synergistic Behaviour of Multiparametric Magnetic Resonance and Prostate Health Index in the Identification of Clinically Significant Prostate Cancer. *Clinical Genitourinary Cancer.* 2022; 20 (5): e406-e410. 10.1016/j.clgc.2022.04.013.

(22) Li H, Lee CH, Chia D, Lin Z, Huang W, Tan CH. Machine Learning in Prostate MRI for Prostate Cancer: Current Status and Future Opportunities. *Diagnostics (Basel, Switzerland).* 2022; 12 (2): 289. 10.3390/diagnostics12020289.

(23) Bulten W, Kartasalo K, Chen PC, Ström P, Pinckaers H, Nagpal K, et al. Artificial intelligence for diagnosis and Gleason grading of prostate cancer: the PANDA challenge. *Nature Medicine.* 2022; 28 (1): 154-163. 10.1038/s41591-021-01620-2.

(24) Aliukonis P, Letauta T, Briedienė R, Naruševičiūtė I, Letautienė S. The role of different PI-RADS versions in prostate multiparametric magnetic resonance tomography assessment. *Acta Medica Lituanica.* 2017; 24 (1): 44-50. 10.6001/actamedica.v24i1.3462.

(25) Moher D, Shamseer L, Clarke M, Ghersi D, Liberati A, Petticrew M, et al. Preferred reporting items for systematic review and meta-analysis protocols (PRISMA-P) 2015 statement. *Systematic Reviews.* 2015; 4 (1): 1. 10.1186/2046-4053-4-1.

(26) Ouzzani M, Hammady H, Fedorowicz Z, Elmagarmid A. Rayyan—a web and mobile app for systematic reviews. *Systematic Reviews.* 2016; 5 10.1186/s13643-016-0384-4.

(27) Whiting PF, Rutjes AWS, Westwood ME, Mallett S, Deeks JJ, Reitsma JB, et al. QUADAS-2: a revised tool for the quality assessment of diagnostic accuracy studies. *Annals of Internal Medicine.* 2011; 155 (8): 529-536. 10.7326/0003-4819-155-8-201110180-00009.

(28) Tang X. The role of artificial intelligence in medical imaging research. *BJR open.* 2020; 2 (1): 20190031. 10.1259/bjro.20190031.

For peer review only

Reporting checklist for protocol of a systematic review and meta analysis.

Based on the PRISMA-P guidelines.

Instructions to authors

Complete this checklist by entering the page numbers from your manuscript where readers will find each of the items listed below.

Your article may not currently address all the items on the checklist. Please modify your text to include the missing information. If you are certain that an item does not apply, please write "n/a" and provide a short explanation.

Upload your completed checklist as an extra file when you submit to a journal.

In your methods section, say that you used the PRISMA-Preporting guidelines, and cite them as:

Moher D, Shamseer L, Clarke M, Ghersi D, Liberati A, Petticrew M, Shekelle P, Stewart LA. Preferred Reporting Items for Systematic Review and Meta-Analysis Protocols (PRISMA-P) 2015 statement. Syst Rev. 2015;4(1):1.

		Reporting Item	Page Number
**Title**			
Identification	#1a	Identify the report as a protocol of a systematic review	1
Update	#1b	If the protocol is for an update of a previous systematic review, identify as such	n/a

For peer review only

**Registration**			
	#2	If registered, provide the name of the registry (such as PROSPERO) and registration number	2
**Authors**			
Contact	#3a	Provide name, institutional affiliation, e-mail address of all protocol authors; provide physical mailing address of corresponding author	1, 10-11
Contribution	#3b	Describe contributions of protocol authors and identify the guarantor of the review	11
**Amendments**			
	#4	If the protocol represents an amendment of a previously completed or published protocol, identify as such and list changes; otherwise, state plan for documenting important protocol amendments	n/a
**Support**			
Sources	#5a	Indicate sources of financial or other support for the review	11
Sponsor	#5b	Provide name for the review funder and / or sponsor	11
Role of sponsor or funder	#5c	Describe roles of funder(s), sponsor(s), and / or institution(s), if any, in developing the protocol	11
**Introduction**			

For peer review only

Rationale	#6	Describe the rationale for the review in the context of what is already known	4
Objectives	#7	Provide an explicit statement of the question(s) the review will address with reference to participants, interventions, comparators, and outcomes (PICO)	5
**Methods**			
Eligibility criteria	#8	Specify the study characteristics (such as PICO, study design, setting, time frame) and report characteristics (such as years considered, language, publication status) to be used as criteria for eligibility for the review	6
Information sources	#9	Describe all intended information sources (such as electronic databases, contact with study authors, trial registers or other grey literature sources) with planned dates of coverage	6
Search strategy	#10	Present draft of search strategy to be used for at least one electronic database, including planned limits, such that it could be repeated	6, 10
Study records - data management	#11a	Describe the mechanism(s) that will be used to manage records and data throughout the review	6-7
Study records - selection process	#11b	State the process that will be used for selecting studies (such as two independent reviewers) through each phase of the review (that is, screening, eligibility and inclusion in meta-analysis)	6

For peer review only

Study records - data collection process	#11c	Describe planned method of extracting data from reports (such as piloting forms, done independently, in duplicate), any processes for obtaining and confirming data from investigators	6
Data items	#12	List and define all variables for which data will be sought (such as PICO items, funding sources), any pre-planned data assumptions and simplifications	7
Outcomes and prioritization	#13	List and define all outcomes for which data will be sought, including prioritization of main and additional outcomes, with rationale	7
Risk of bias in individual studies	#14	Describe anticipated methods for assessing risk of bias of individual studies, including whether this will be done at the outcome or study level, or both; state how this information will be used in data synthesis	8
Data synthesis	#15a	Describe criteria under which study data will be quantitatively synthesised	7-8
Data synthesis	#15b	If data are appropriate for quantitative synthesis, describe planned summary measures, methods of handling data and methods of combining data from studies, including any planned exploration of consistency (such as I2, Kendall's τ)	7-8
Data synthesis	#15c	Describe any proposed additional analyses (such as sensitivity or subgroup analyses, meta-regression)	7-8

For peer review only

Data synthesis	#15d	If quantitative synthesis is not appropriate, describe the type of summary planned	7-8
Meta-bias(es)	#16	Specify any planned assessment of meta-bias(es) (such as publication bias across studies, selective reporting within studies)	8
Confidence in cumulative evidence	#17	Describe how the strength of the body of evidence will be assessed (such as GRADE)	8

The PRISMA-P elaboration and explanation paper is distributed under the terms of the Creative Commons Attribution License CC-BY. This checklist was completed on 20. March 2023 using https://www.goodreports.org/, a tool made by the EQUATOR Network in collaboration with Penelope.ai

For peer review only

BMJ Open

The use of artificial intelligence in the detection of primary prostate cancer in multiparametric magnetic resonance imaging with its clinical outcomes: a protocol for a systematic review and meta-analysis

Journal:	*BMJ Open*
Manuscript ID	bmjopen-2023-074009.R1
Article Type:	Protocol
Date Submitted by the Author:	11-Jul-2023
Complete List of Authors:	Thomas, Maya; Imperial College London, School of Medicine<br>Murali, Sanjana; Imperial College London, School of Medicine<br>Simpson, Benjamin Scott; University College London, UCL Cancer Institute<br>Freeman, Alex; University College London Hospitals NHS Foundation Trust, Department of Pathology<br>Kirkham, Alex; University College London Hospitals NHS Foundation Trust, Department of Radiology<br>Kelly, Daniel; Cardiff University, School of Healthcare Sciences<br>Whitaker, Hayley; University College London, UCL Division of Surgery and Interventional Science<br>Zhao, Yi; Imperial College London, School of Medicine<br>Emberton, Mark; University College London, UCL Division of Surgery & Interventional Science; University College London Hospitals NHS Foundation Trust, Department of Urology<br>Norris, Joseph; University College London, UCL Division of Surgery & Interventional Science; University College London Hospitals NHS Foundation Trust, Department of Urology
**Primary Subject Heading**:	Urology
Secondary Subject Heading:	Radiology and imaging, Oncology
Keywords:	Prostate, Urological tumours < UROLOGY, Urological tumours < ONCOLOGY, RADIOLOGY & IMAGING, Magnetic resonance imaging < RADIOLOGY & IMAGING

SCHOLARONE™
Manuscripts

The use of artificial intelligence in the detection of primary prostate cancer in multiparametric magnetic resonance imaging with its clinical outcomes: a protocol for a systematic review and meta-analysis

Maya Thomas[1], Sanjana Murali[1], Benjamin S. Simpson[2], Alex Freeman[3], Alex Kirkham[4], Daniel Kelly[5], Hayley C. Whitaker[6], Yi Zhao[1], Mark Emberton[6,7], Joseph M. Norris[6,7]

MT and SM are joint first authors.

ME and JMN are joint senior authors.

[1] Imperial College London School of Medicine, London, UK

[2] UCL Cancer Institute, University College London, London, UK

[3] Department of Pathology, University College London Hospitals NHS Foundation Trust, London, UK

[4] Department of Radiology, University College London Hospitals NHS Foundation Trust, London, UK

[5] School of Healthcare Sciences, Cardiff University, Wales, UK

[6] UCL Division of Surgery & Interventional Science, University College London, London, UK

[7] Department of Urology, University College London Hospitals NHS Foundation Trust, London, UK

Correspondence to: maya.thomas18@imperial.ac.uk

For peer review only

Imperial College London

Exhibition Rd, South Kensington, London SW7 2BU

maya.thomas18@imperial.ac.uk

Ethics: No ethical considerations apply.

Word count: 1924

For peer review only

ABSTRACT

Introduction: Multiparametric magnetic resonance imaging (mpMRI) has transformed the prostate cancer diagnostic pathway, allowing for improved risk stratification and more targeted subsequent management. However, concerns exist over the interobserver variability of images and the applicability of this model long term, especially considering the current shortage of radiologists and the growing ageing population. Artificial intelligence (AI) is being integrated into clinical practice to support diagnostic and therapeutic imaging analysis to overcome these concerns. The following report details a protocol for a systematic review and meta-analysis investigating the accuracy of AI in predicting primary prostate cancer on mpMRI.

Methods and Analysis: A systematic search will be performed using PubMed, MEDLINE, Embase, and Cochrane databases. All relevant articles published between January 2016 and February 2023 will be eligible for inclusion. To be included, articles must use AI to study MRI prostate images to detect prostate cancer. All included articles will be in full-text, reporting original data, and written in English. The protocol follows the Preferred Reporting Items for Systematic Review and Meta-Analysis Protocol (PRISMA-P) 2015 checklist. The QUADAS-2 score will assess the quality and risk of bias across selected studies.

Ethics and dissemination: Ethical approval will not be required for this systematic review. Findings will be disseminated through peer-reviewed publications and presentations at both national and international conferences.

PROSPERO registration number: CRD42021293745

For peer review only

Strengths and limitations of this study:

- This paper hopes to provide a comprehensive systematic review and meta-analysis which explores in depth the multitude of AI algorithms utilised for prostate multiparametric MRI in line with PRISMA guidelines.
- AI-specific metrics such as the F1-score and precision-recall AUC will be employed to mitigate the restrictions of conventional pooled analysis, such as the impact of class imbalance.
- As AI is a relatively novel technology in mpMRI, the long-term data on clinical outcomes associated with its use may be limited.
- The restrictions on language and use of multiparametric MRI only may result in few suitable studies for inclusion.

For peer review only

BACKGROUND

Accurately identifying clinically significant prostate cancer has been a long-standing, challenging issue. Previous diagnostic pathways have resulted in many patients undergoing biopsies diagnosed with clinically insignificant prostate cancer. This can lead to potentially aggressive overtreatment, unnecessary healthcare cost and detrimental effects on patient mental health[1-3].

The introduction of multiparametric magnetic resonance imaging (mpMRI) was pivotal in resolving this issue. Multiple sources now recommend using mpMRI as a prebiopsy investigation[2-7] to allow earlier risk stratification of those with suspected prostate cancer and improve the utility of the biopsies performed by enabling a more targeted approach. Despite this, interobserver variability of images remains a major concern[5,8], with a lack of standardised protocols and a requirement for experienced radiologists being examples of contributory factors[4,9]. While radiology trainees show rapid improvement

For peer review only

in the accuracy of interpreting studies with increased exposure, there is still a margin of error at around 25%[10]. Additionally, prostate cancer is the second most frequent cancer diagnosis globally, and the healthcare burden is only set to increase due to a growing ageing population. Therefore, the volume of diagnostic imaging and management monitoring required of radiologists will also likely increase over time[11].

Artificial Intelligence (AI), including Deep Learning (DL), describes how computers can be programmed to imitate human intelligence[12]. AI can be utilised in a semiautomated or fully automated approach. Semiautomated AI refers to using more traditional machine learning mechanisms such as radiomics, where radiologists must perform some pre-processing of images to make them algorithm appropriate. Fully automated AI has the benefit of using neural networks to perform feature identification[13]. Neural networks are a subset of deep learning models that mimic the human visual cortex[14]. Each neural network layer comprises neurons that identify several features of an image by applying multiple edge, colour and texture filters[15]. These neural networks are trained to associate specific characteristics with certain classifications by iterating through a training dataset of images. These trained layers can work together to perform feature identification on unseen photos; for example, in mpMRI, they could segment a potentially suspicious lesion in the prostate[16,17]. AI is being integrated into clinical practice to support diagnoses, assist in therapeutic decisions, and predict patient outcomes[18]. Several studies have assessed the level of agreement between AI and DL to clinical radiologists to interpret mpMRI in

For peer review only

prostate cancer investigation[19-22]. Application of AI could improve the current interobserver variability, expedite mpMRI interpretation[21], and risk-stratify potentially suspicious lesions for radiologist review.

However, it is also essential to address that DL algorithms are not yet advanced enough for unsupervised clinical utilisation[19,23]. Many issues surround the training of AI models, such as lack of diversity in training datasets – primarily in ethnicity, with many cohorts using predominantly white patients[24], even though prostate cancer is more prevalent and aggressive in Afro-Caribbean men[11]. In many studies, there is a lack of external validation, which makes it unclear how models would perform on new data, and also runs the risk of overfitting to training data – where the model is excellent at recognising lesions in training data but is unable to recognise lesions in new data[24]. Class imbalance due to most negative cases can also present as an issue by creating algorithm bias and false negatives, which warrants review by senior radiologists[24].

Furthermore, variations of the Prostate Imaging Reporting and Data System (PI-RADS) exist – with version 1 introduced in 2011, replaced by version 2 in 2016, and version 2.1 in 2019[25,26]. This makes evaluating how comparable the AI is more challenging due to a lack of consistency. This study will explore this in more detail to inform future reporting methodologies if different PI-RADS versions are used in the included studies.

For peer review only

This systematic review and meta-analysis aims to summarise the accuracy of AI in predicting prostate cancer on mpMRI and how its use may impact clinical outcomes in patients.

METHODS AND ANALYSIS

The protocol for this systematic review follows the Preferred Reporting Items for Systematic Review and Meta-Analysis Protocols (PRISMA-P) 2015 checklist[27]. The study has been prospectively registered with PROSPERO review databases (CRD42021293745), and all methods described here were established before implementation. The statistical data evaluating the sensitivity, specificity, positive predictive value (PPV), negative predictive value (NPV), and area-under-the-curve (AUC) of the use of AI in mpMRI prostate imaging for the detection of prostate cancer will be derived following a thorough analysis and thematic synthesis of included studies. These studies' pooled sensitivities and specificities will be determined before PPV and NPV values are derived.

Search Methodology

A systematic search will be performed using PubMed, MEDLINE, Embase and Cochrane databases. The search strategy will include using medical subject heading (MeSH) terms and free text with appropriate Boolean operators and will encompass articles from January 2016 to February 2023 to achieve maximum yield of the relevant evidence. The search will include the following key terms: "prostate", "cancer", "diagnosis", and multiple synonyms for "mpMRI", "artificial intelligence", and "deep learning". The full search strategy is detailed in supplementary file 1. To facilitate the initial screening

For peer review only

process, Rayyan will be employed, a semiautomated application designed to improve the speed and reporting accuracy of systematic reviews[28]. All eligible articles from the initial search will be uploaded to Rayyan. A further manual search of the references in all included articles will be performed to identify any further relevant literature not found by the initial search strategy. If data is absent or ambiguous, the corresponding authors will be contacted for clarification.

Study selection and data extraction

The screening process will be completed independently by two researchers (M.T. and S.M.). Titles and abstracts of eligible studies will be assessed, and irrelevant articles will be removed. A full-text version of relevant articles will be downloaded for further eligibility review. Any dispute amongst researchers will be discussed, and a third reviewer (Y.Z.) will be consulted, with the issue being resolved by consensus. The reasoning for excluding articles will be documented and detailed in a PRISMA flow diagram. Before commencing the screening process, calibration exercises will be conducted to maintain consistency between the researchers, reducing inter-reviewer bias.

Inclusion and exclusion criteria

For inclusion in this systematic review, studies must use either fully automated or semiautomated AI to study multiparametric MRI prostate images to detect prostate cancer. Prospective or retrospective studies can be included. Comparisons will be focussed on evaluating sensitivities, specificities, PPVs,

For peer review only

NPVs and AUCs. Patient cohorts can comprise suspected and confirmed incidences of cancer, and studies included should include reference standards using histological findings.

Correspondence papers, ongoing studies, case reports and conference abstracts reports will be excluded from this analysis. Articles which are not written in the English Language will also be excluded. Papers not using multiparametric MRI as the diagnostic modality will be excluded. Studies which include patients with previous treatment of prostate cancer will be excluded.

Data extraction (table of collection)

The following data detailed in Table 1 will be collected from all included studies. The researchers will perform data extraction independently, collating collected data in a dedicated datasheet. Any discrepancies in data extraction will be examined by a third reviewer and will be resolved by consensus.

If the data is available, the relevant figures will be extracted appropriately, including but not limited to true positive, true negative, false positive and false negatives and derivatives of these calculations. If these are not provided, then attempts will be made to calculate from the data provided. If this is unsuccessful, the relevant authors of the paper in question will be contacted to provide the data.

Table 1. Data collection items

**ITEM NO.**	DATA TITLE	DATA TYPE
**1**	Year of publication	Study characteristic

For peer review only

**2**	Study authors	Study characteristic
**3**	Experimental design	Study characteristic
**4**	Patient population	Demographics
**5**	Study size	Demographics
**6**	Type of MRI imaging used	Methodology
**7**	Type of MRI scoring system used	Methodology
**8**	Type of AI models used	Methodology
**9**	Definition of clinically significant disease	Methodology
**10**	Predictive performances	Outcome

AI: artificial intelligence; MRI: magnetic resonance imaging

Endpoints

The primary endpoint will be statistically significant quantified accuracy in using AI in MRI prostate imaging to detect prostate cancer and determine whether AI could be deployed in the clinical decision-making process. Additional outcomes will include other parameters investigating the extent of the disease.

Meta-analysis

A meta-analysis will be conducted if there are a sufficient number of appropriate studies. This would aim to obtain a pooled quantitative diagnostic accuracy value for AI performance detecting prostate

For peer review only

cancer from mpMRI. To begin, sensitivity and specificity values will be retrieved, or if studies do not provide these values, they will be calculated from clinical tables or requested from corresponding authors. If a considerable proportion of included studies used another metric, these values would be retrieved and calculated separately. This may include common AI-focused metrics such as F1-score and precision-recall AUC. The distributions of the untransformed, logit and double-arcsine transformed proportions will be compared and assessed for normality using density plots and Shapiro-Wilk tests, with whichever set of ratios most resembling normal distribution being used for further analysis.

Inter-study variation will be quantified through $I^{2,}$ and if statistically significant, a random-effects model will be fitted for estimation of the summary estimate. Once the model fits all relevant studies, leave-one-out analysis (LOO) and accompanying diagnostic plots will be used to identify influential studies. This will include externally studentised residuals, the difference in fits values (DFFITS), Cook's distances, covariance ratios, LOO estimates of the amount of heterogeneity, LOO values of the test statistics for heterogeneity, hat values and weights. Each study will be removed one at a time, and the summary proportions re-estimated based on the remaining n-1 studies. Studies with a statistically significant influence on the fitted model will be considered outliers and removed. The model will then be re-fitted. A summary estimate comprising the remaining studies will then be calculated to estimate the accuracy using AI in mpMRI for prostate cancer detection. All data analysis and visualisation will be performed using the R statistical environment using the "mvmeta" and "meta" packages.

For peer review only

Risk of bias in individual studies

The QUADAS-2 score will be employed to assess the quality and the risk of bias across the selected studies[29]. This tool comprises four domains: patient selection, index test, reference standard, and flow and timing. The quality of the research methodology for each study will be assessed within these four domains. Two (M.T. and Y.Z.) reviewers will be independently involved in this process, with any disparities resolved by consensus. The bias assessment will evaluate the applicability and reliability of the data used. This will inform evidence synthesis and increase transparency as articles may be excluded if found to be of low quality or suggestive of high levels of bias, or if included, appropriate commentaries will be incorporated into the discussion.

Ethics and Dissemination

AI and DL have substantial roles in streamlining service provision and optimising workloads for radiologists, however, the potential role of AI in assisting mpMRI interpretation for prostate cancer diagnosis is yet to be evaluated. Through the summation of this information, we hope to clarify the role of AI in improving reporting and summarise its overall impact on clinical care.

This study will systematically collect, analyse and report the clinical studies available to better inform the potential of AI and DL in the diagnosis of prostate cancer. However, several limitations should be highlighted. First, we recognise that due to its relative novelty, there may be a limited number of studies that have evaluated AI in this context. Next, there could be issues surrounding the heterogeneity of

For peer review only

reporting protocols due to local variation. Finally, different neural networks may also act as confounders due to different training datasets, which would arguably report differently depending on learned features.

We will publish the results in a peer-reviewed journal. The synthesis of this data will result in a better understanding of current AI and DL protocols and how existing systems can be further improved for supervised and unsupervised usage. We envisage this process will highlight areas requiring further research and will help optimise reporting protocols in the future, therefore aiding prostate cancer diagnosis and improving patient outcomes.

Patient and public involvement

There will be no patient or public involvement in this study.

Trial status:

- Preliminary searches: Started
- Piloting of the study selection process: Started
- Formal screening: Not started
- Data extraction: Not started
- Risk of bias assessment: Not started
- Data analysis: Not started

For peer review only

Author Affiliations

[1] Imperial College London School of Medicine, London, UK

[2] UCL Cancer Institute, University College London, London, UK

[3] Department of Pathology, University College London Hospitals NHS Foundation Trust, London, UK

[4] Department of Radiology, University College London Hospitals NHS Foundation Trust, London, UK

[5] School of Healthcare Sciences, Cardiff University, Wales, UK

[6] UCL Division of Surgery & Interventional Science, University College London, London, UK

[7] Department of Urology, University College London Hospitals NHS Foundation Trust, London, UK

Email: Maya Thomas: maya.thomas18@imperial.ac.uk, Sanjana Murali: sanjana.murali18@imperial.ac.uk, Yi Zhao: yi.zhao18@imperial.ac.uk, Benjamin S. Simpson: b.simpson@ucl.ac.uk, Alex Freeman: alex.freeman2@nhs.net, Alex Kirkam: alexkirkham@nhs.net, Daniel Kelly: kellydm@cardiff.ac.uk, Hayley C. Whitaker: hayley.whitaker@ucl.ac.uk, Mark Emberton: m.emberton@ucl.ac.uk, Joseph M. Norris: joseph.norris@ucl.ac.uk

Twitter Benjamin S. Simpson @b3nsimp, Daniel Kelly @profdkelly, Hayley C. Whitaker @HayleyWhitaker, Mark Emberton @EmbertonMark, Joseph M. Norris @MrJosephNorris

For peer review only

Contributors The authors' contribution includes, but is not limited to, the following: MT and SM wrote the manuscript. YZ and JMN created the study concept. YZ, BSS, AF, AK, DK, HCW and ME provided supervision and guidance. All authors reviewed and approved the manuscript in its current form. MT and SM are the guarantors of this work.

Funding Norris is funded by the Medical Research Council (MRC) (MR/S00680X/1). Simpson is funded by the Royal Marsden Cancer Charity. Emberton receives research support from the United Kingdom's National Institute of Health Research (NIHR) UCLH/UCL Biochemical Research Centre.

Competing Interests Norris receives funding from the MRC. Simpson receives funding from the Royal Marsden Cancer Charity. Whitaker receives funding from Prostate Cancer UK, the Urology Foundation and Rosetrees Trust. Kirkham, Freeman and Emberton have stock interest in Nuada Medical Ltd. Emberton acts as a consultant, trainer and proctor to Sonatherm Inc; Angiodynamics Inc; Exact Imaging Inc.

Patient consent for publication Not required

Provenance and peer review Not commissioned; externally peer-reviewed

Open access This is an open-access article distributed in accordance with the Creative Commons Attribution 4.0 Unreported (CC BY 4.0) license which permits others to copy, redistribute, remix, transform and build upon this work for any purpose, provided the original work is properly cited, a link to the licence is given, and an indication of whether changes were made. See https://creativecommons.org/licenses/by/4.0/.

For peer review only

ORCID IDs

- Sanjana Murali https://orcid.org/0000-0001-7980-6733
- Maya Thomas https://orcid.org/0000-0001-6997-9438
- Yi Zhao https://orcid.org/0000-0002-4563-4344
- Naomi Morka https://orcid.org/0000-0002-1925-7223
- Joseph M. Norris http://orcid.org/0000-0003-2294-0303
- Benjamin S. Simpson http://orcid.org/0000-0003-3685-6110
- Daniel Kelly http://orcid.org/0000-0002-1847-0655
- Hayley C. Whitaker https://orcid.org/0000-0002-2695-0202

References

(1) Sandhu GS, Andriole GL. Overdiagnosis of Prostate Cancer. *Journal of the National Cancer Institute. Monographs.* 2012; 2012 (45): 146-151. 10.1093/jncimonographs/lgs031.

(2) Kasivisvanathan V, Rannikko AS, Borghi M, Panebianco V, Mynderse LA, Vaarala MH, et al. MRI-Targeted or Standard Biopsy for Prostate-Cancer Diagnosis. *New England Journal of Medicine.* 2018; 378 (19): 1767-1777. 10.1056/NEJMoa1801993.

(3) Brown LC, Ahmed HU, Faria R, El-Shater Bosaily A, Gabe R, Kaplan RS, et al. Multiparametric MRI to improve detection of prostate cancer compared with transrectal ultrasound-guided prostate biopsy alone: the PROMIS study. *Health Technology Assessment.* 2018; 22 (39): 1-176. 10.3310/hta22390.

(4) Zhen L, Liu X, Yegang C, Yongjiao Y, Yawei X, Jiaqi K, et al. Accuracy of multiparametric magnetic resonance imaging for diagnosing prostate Cancer: a systematic review and meta-analysis. *BMC cancer.* 2019; 19 (1): 1244. 10.1186/s12885-019-6434-2.

(5) Richenberg J, Logager V, Panebianco V, Rouviere O, Villeirs G, Schoots I. The primacy of multiparametric MRI in men with suspected prostate cancer. *European Radiology.* 2019; 29 (12): 6940-6952. 10.1007/s00330-019-06166-z.

(6) van der Leest M, Cornel E, Israël B, Hendriks R, Padhani AR, Hoogenboom M, et al. Head-to-head Comparison of Transrectal Ultrasound-guided Prostate Biopsy Versus Multiparametric Prostate Resonance Imaging with Subsequent Magnetic Resonance-guided Biopsy in Biopsy-naïve Men with Elevated Prostate-

For peer review only

specific Antigen: A Large Prospective Multicenter Clinical Study. *European Urology.* 2019; 75 (4): 570-578. 10.1016/j.eururo.2018.11.023.

(7) Ahmed HU, El-Shater Bosaily A, Brown LC, Gabe R, Kaplan R, Parmar MK, et al. Diagnostic accuracy of multiparametric MRI and TRUS biopsy in prostate cancer (PROMIS): a paired validating confirmatory study. *Lancet (London, England).* 2017; 389 (10071): 815-822. 10.1016/S0140-6736(16)32401-1.

(8) Stabile A, Giganti F, Rosenkrantz AB, Taneja SS, Villeirs G, Gill IS, et al. Multiparametric MRI for prostate cancer diagnosis: current status and future directions. *Nature Reviews. Urology.* 2020; 17 (1): 41-61. 10.1038/s41585-019-0212-4.

(9) Dominguez C, Plata M, Cataño JG, Palau M, Aguirre D, Narvaez J, et al. Diagnostic accuracy of multiparametric magnetic resonance imaging in detecting extracapsular extension in intermediate and high-risk prostate cancer. *International Brazilian Journal of Urology : official journal of the Brazilian Society of Urology.* 2018; 44 (4): 688-696. 10.1590/S1677-5538.IBJU.2016.0485.

(10) Rosenkrantz AB, Ayoola A, Hoffman D, Khasgiwala A, Prabhu V, Smereka P, et al. The Learning Curve in Prostate MRI Interpretation: Self-Directed Learning Versus Continual Reader Feedback. *AJR. American journal of roentgenology.* 2017; 208 (3): W92-W100. 10.2214/AJR.16.16876.

(11) Rawla P. Epidemiology of Prostate Cancer. *World Journal of Oncology.* 2019; 10 (2): 63-89. 10.14740/wjon1191.

(12) Gore JC. Artificial intelligence in medical imaging. *Magnetic resonance imaging.* 2020; 68 A1-A4. 10.1016/j.mri.2019.12.006.

(13) Sushentsev N, Moreira Da Silva N, Yeung M, Barrett T, Sala E, Roberts M, et al. Comparative performance of fully-automated and semiautomated artificial intelligence methods for the detection of clinically significant prostate cancer on MRI: a systematic review. *Insights into Imaging.* 2022; 13 (1): 59. 10.1186/s13244-022-01199-3.

(14) van Dyck LE, Kwitt R, Denzler SJ, Gruber WR. Comparing Object Recognition in Humans and Deep Convolutional Neural Networks—An Eye Tracking Study. *Frontiers in Neuroscience.* 2021; 15 750639. 10.3389/fnins.2021.750639.

(15) Saha A, Fadaiefard P, Rabski J, Sadeghian A, Cusimano M. *Machine learning applications using diffusion tensor imaging of human brain: A PubMed literature review.* ; 2020.

(16) Kriegeskorte N, Golan T. Neural network models and deep learning. *Current biology: CB.* 2019; 29 (7): R231-R236. 10.1016/j.cub.2019.02.034.

(17) Schelb P, Kohl S, Radtke JP, Wiesenfarth M, Kickingereder P, Bickelhaupt S, et al. Classification of Cancer at Prostate MRI: Deep Learning versus Clinical PI-RADS Assessment. *Radiology.* 2019; 293 (3): 607-617. 10.1148/radiol.2019190938.

For peer review only

(18) Briganti G, Le Moine O. Artificial Intelligence in Medicine: Today and Tomorrow. *Frontiers in Medicine.* 2020; 7 27. 10.3389/fmed.2020.00027.

(19) Syer T, Mehta P, Antonelli M, Mallett S, Atkinson D, Ourselin S, et al. Artificial Intelligence Compared to Radiologists for the Initial Diagnosis of Prostate Cancer on Magnetic Resonance Imaging: A Systematic Review and Recommendations for Future Studies. *Cancers.* 2021; 13 (13): 3318. 10.3390/cancers13133318.

(20) Van Booven DJ, Kuchakulla M, Pai R, Frech FS, Ramasahayam R, Reddy P, et al. A Systematic Review of Artificial Intelligence in Prostate Cancer. *Research and Reports in Urology.* 2021; 13 31-39. 10.2147/RRU.S268596.

(21) Soerensen SJC, Fan RE, Seetharaman A, Chen L, Shao W, Bhattacharya I, et al. Deep Learning Improves Speed and Accuracy of Prostate Gland Segmentations on Magnetic Resonance Imaging for Targeted Biopsy. *The Journal of Urology.* 2021; 206 (3): 604-612. 10.1097/JU.0000000000001783.

(22) Gentile F, La Civita E, Della Ventura B, Ferro M, Cennamo M, Bruzzese D, et al. A Combinatorial Neural Network Analysis Reveals a Synergistic Behaviour of Multiparametric Magnetic Resonance and Prostate Health Index in the Identification of Clinically Significant Prostate Cancer. *Clinical Genitourinary Cancer.* 2022; 20 (5): e406-e410. 10.1016/j.clgc.2022.04.013.

(23) Li H, Lee CH, Chia D, Lin Z, Huang W, Tan CH. Machine Learning in Prostate MRI for Prostate Cancer: Current Status and Future Opportunities. *Diagnostics (Basel, Switzerland).* 2022; 12 (2): 289. 10.3390/diagnostics12020289.

(24) Bulten W, Kartasalo K, Chen PC, Ström P, Pinckaers H, Nagpal K, et al. Artificial intelligence for diagnosis and Gleason grading of prostate cancer: the PANDA challenge. *Nature Medicine.* 2022; 28 (1): 154-163. 10.1038/s41591-021-01620-2.

(25) Aliukonis P, Letauta T, Briedienė R, Naruševičiūtė I, Letautienė S. The role of different PI-RADS versions in prostate multiparametric magnetic resonance tomography assessment. *Acta Medica Lituanica.* 2017; 24 (1): 44-50. 10.6001/actamedica.v24i1.3462.

(26) Turkbey B, Rosenkrantz AB, Haider MA, Padhani AR, Villeirs G, Macura KJ, et al. Prostate Imaging Reporting and Data System Version 2.1: 2019 Update of Prostate Imaging Reporting and Data System Version 2. *European Urology.* 2019; 76 (3): 340-351. 10.1016/j.eururo.2019.02.033.

(27) Moher D, Shamseer L, Clarke M, Ghersi D, Liberati A, Petticrew M, et al. Preferred reporting items for systematic review and meta-analysis protocols (PRISMA-P) 2015 statement. *Systematic Reviews.* 2015; 4 (1): 1. 10.1186/2046-4053-4-1.

(28) Ouzzani M, Hammady H, Fedorowicz Z, Elmagarmid A. Rayyan—a web and mobile app for systematic reviews. *Systematic Reviews.* 2016; 5 10.1186/s13643-016-0384-4.

For peer review only

389 (29) Whiting PF, Rutjes AWS, Westwood ME, Mallett S, Deeks JJ, Reitsma JB, et al. QUADAS-2: a revised tool for the quality assessment of diagnostic accuracy studies. *Annals of Internal Medicine.* 2011; 155 (8): 529-536. 10.7326/0003-4819-155-8-201110180-00009.

For peer review only

The use of artificial intelligence in the detection of primary prostate cancer in multiparametric magnetic resonance imaging with its clinical outcomes: a protocol for a systematic review and meta-analysis

The search terms for this systematic review are as follows:

((artificial intelligen* OR machine intelligen* OR computational intelligen* OR neural network OR deep learning OR machine learning OR reinforcement learning OR convolutional neural network OR artificial neural network OR recurrent neural network OR deep neural network OR intelligent model OR AI OR NN OR DL OR ML OR CNN OR ANN OR RNN OR DNN) AND ((Prostat* AND (cancer OR malignan* OR adenocarcinoma OR lesion* OR disease)) AND (MR OR magnetic resonance imaging OR MP-MRI OR multi-parametric MRI OR multi-parametric magnetic resonance imaging OR multiparametric MRI OR “multiparametric magnetic resonance imaging” OR radiomic* OR radiogenomic*)) AND diagnosis).ti,ab

Searches will be conducted on the following databases:

PubMed, MEDLINE, Embase, and Cochrane databases

For peer review only

Reporting checklist for protocol of a systematic review and meta analysis.

Based on the PRISMA-P guidelines.

Instructions to authors

Complete this checklist by entering the page numbers from your manuscript where readers will find each of the items listed below.

Your article may not currently address all the items on the checklist. Please modify your text to include the missing information. If you are certain that an item does not apply, please write "n/a" and provide a short explanation.

Upload your completed checklist as an extra file when you submit to a journal.

In your methods section, say that you used the PRISMA-Preporting guidelines, and cite them as:

Moher D, Shamseer L, Clarke M, Ghersi D, Liberati A, Petticrew M, Shekelle P, Stewart LA. Preferred Reporting Items for Systematic Review and Meta-Analysis Protocols (PRISMA-P) 2015 statement. Syst Rev. 2015;4(1):1.

		Reporting Item	Page Number
**Title**			
Identification	#1a	Identify the report as a protocol of a systematic review	1
Update	#1b	If the protocol is for an update of a previous systematic review, identify as such	n/a

For peer review only

Registration

	#2	If registered, provide the name of the registry (such as PROSPERO) and registration number	2

Authors

Contact	#3a	Provide name, institutional affiliation, e-mail address of all protocol authors; provide physical mailing address of corresponding author	1, 10-11
Contribution	#3b	Describe contributions of protocol authors and identify the guarantor of the review	11

Amendments

	#4	If the protocol represents an amendment of a previously completed or published protocol, identify as such and list changes; otherwise, state plan for documenting important protocol amendments	n/a

Support

Sources	#5a	Indicate sources of financial or other support for the review	11
Sponsor	#5b	Provide name for the review funder and / or sponsor	11
Role of sponsor or funder	#5c	Describe roles of funder(s), sponsor(s), and / or institution(s), if any, in developing the protocol	11

Introduction

For peer review only

Rationale	#6	Describe the rationale for the review in the context of what is already known	4
Objectives	#7	Provide an explicit statement of the question(s) the review will address with reference to participants, interventions, comparators, and outcomes (PICO)	5, 6-7
**Methods**			
Eligibility criteria	#8	Specify the study characteristics (such as PICO, study design, setting, time frame) and report characteristics (such as years considered, language, publication status) to be used as criteria for eligibility for the review	6-7
Information sources	#9	Describe all intended information sources (such as electronic databases, contact with study authors, trial registers or other grey literature sources) with planned dates of coverage	6-7
Search strategy	#10	Present draft of search strategy to be used for at least one electronic database, including planned limits, such that it could be repeated	Supplementary file
Study records - data management	#11a	Describe the mechanism(s) that will be used to manage records and data throughout the review	6-7
Study records - selection process	#11b	State the process that will be used for selecting studies (such as two independent reviewers) through	6-7

For peer review only

		each phase of the review (that is, screening, eligibility and inclusion in meta-analysis)	
Study records - data collection process	#11c	Describe planned method of extracting data from reports (such as piloting forms, done independently, in duplicate), any processes for obtaining and confirming data from investigators	7
Data items	#12	List and define all variables for which data will be sought (such as PICO items, funding sources), any pre-planned data assumptions and simplifications	7
Outcomes and prioritization	#13	List and define all outcomes for which data will be sought, including prioritization of main and additional outcomes, with rationale	7-8
Risk of bias in individual studies	#14	Describe anticipated methods for assessing risk of bias of individual studies, including whether this will be done at the outcome or study level, or both; state how this information will be used in data synthesis	9
Data synthesis	#15a	Describe criteria under which study data will be quantitatively synthesised	8
Data synthesis	#15b	If data are appropriate for quantitative synthesis, describe planned summary measures, methods of handling data and methods of combining data from studies, including any planned exploration of consistency (such as I2, Kendall's τ)	8

For peer review only

Data synthesis	#15c	Describe any proposed additional analyses (such as sensitivity or subgroup analyses, meta-regression)	8
Data synthesis	#15d	If quantitative synthesis is not appropriate, describe the type of summary planned	8
Meta-bias(es)	#16	Specify any planned assessment of meta-bias(es) (such as publication bias across studies, selective reporting within studies)	8-9
Confidence in cumulative evidence	#17	Describe how the strength of the body of evidence will be assessed (such as GRADE)	8-9

The PRISMA-P elaboration and explanation paper is distributed under the terms of the Creative Commons Attribution License CC-BY. This checklist was completed on 20. March 2023 using https://www.goodreports.org/, a tool made by the EQUATOR Network in collaboration with Penelope.ai

For peer review only

BMJ Open

The use of artificial intelligence in the detection of primary prostate cancer in multiparametric magnetic resonance imaging with its clinical outcomes: a protocol for a systematic review and meta-analysis

Journal:	*BMJ Open*
Manuscript ID	bmjopen-2023-074009.R2
Article Type:	Protocol
Date Submitted by the Author:	24-Jul-2023
Complete List of Authors:	Thomas, Maya; Imperial College London, School of Medicine<br>Murali, Sanjana; Imperial College London, School of Medicine<br>Simpson, Benjamin Scott; University College London, UCL Cancer Institute<br>Freeman, Alex; University College London Hospitals NHS Foundation Trust, Department of Pathology<br>Kirkham, Alex; University College London Hospitals NHS Foundation Trust, Department of Radiology<br>Kelly, Daniel; Cardiff University, School of Healthcare Sciences<br>Whitaker, Hayley; University College London, UCL Division of Surgery and Interventional Science<br>Zhao, Yi; Imperial College London, School of Medicine<br>Emberton, Mark; University College London, UCL Division of Surgery & Interventional Science; University College London Hospitals NHS Foundation Trust, Department of Urology<br>Norris, Joseph; University College London, UCL Division of Surgery & Interventional Science; University College London Hospitals NHS Foundation Trust, Department of Urology
**Primary Subject Heading**:	Urology
Secondary Subject Heading:	Radiology and imaging, Oncology
Keywords:	Prostate, Urological tumours < UROLOGY, Urological tumours < ONCOLOGY, RADIOLOGY & IMAGING, Magnetic resonance imaging < RADIOLOGY & IMAGING

SCHOLARONE™
Manuscripts

The use of artificial intelligence in the detection of primary prostate cancer in multiparametric magnetic resonance imaging with its clinical outcomes: a protocol for a systematic review and meta-analysis

Maya Thomas[1], Sanjana Murali[1], Benjamin S. Simpson[2], Alex Freeman[3], Alex Kirkham[4], Daniel Kelly[5], Hayley C. Whitaker[6], Yi Zhao[1], Mark Emberton[6,7], Joseph M. Norris[6,7]

MT and SM are joint first authors.

ME and JMN are joint senior authors.

[1] Imperial College London School of Medicine, London, UK

[2] UCL Cancer Institute, University College London, London, UK

[3] Department of Pathology, University College London Hospitals NHS Foundation Trust, London, UK

[4] Department of Radiology, University College London Hospitals NHS Foundation Trust, London, UK

[5] School of Healthcare Sciences, Cardiff University, Wales, UK

[6] UCL Division of Surgery & Interventional Science, University College London, London, UK

[7] Department of Urology, University College London Hospitals NHS Foundation Trust, London, UK

Correspondence to: maya.thomas18@imperial.ac.uk

For peer review only

Imperial College London

Exhibition Rd, South Kensington, London SW7 2BU

maya.thomas18@imperial.ac.uk

Ethics: No ethical considerations apply.

Word count: 1908

For peer review only

ABSTRACT

Introduction: Multiparametric magnetic resonance imaging (mpMRI) has transformed the prostate cancer diagnostic pathway, allowing for improved risk stratification and more targeted subsequent management. However, concerns exist over the interobserver variability of images and the applicability of this model long term, especially considering the current shortage of radiologists and the growing ageing population. Artificial intelligence (AI) is being integrated into clinical practice to support diagnostic and therapeutic imaging analysis to overcome these concerns. The following report details a protocol for a systematic review and meta-analysis investigating the accuracy of AI in predicting primary prostate cancer on mpMRI.

Methods and Analysis: A systematic search will be performed using PubMed, MEDLINE, Embase, and Cochrane databases. All relevant articles published between January 2016 and February 2023 will be eligible for inclusion. To be included, articles must use AI to study MRI prostate images to detect prostate cancer. All included articles will be in full-text, reporting original data, and written in English. The protocol follows the Preferred Reporting Items for Systematic Review and Meta-Analysis Protocol (PRISMA-P) 2015 checklist. The QUADAS-2 score will assess the quality and risk of bias across selected studies.

Ethics and dissemination: Ethical approval will not be required for this systematic review. Findings will be disseminated through peer-reviewed publications and presentations at both national and international conferences.

PROSPERO registration number: CRD42021293745

For peer review only

Strengths and limitations of this study:

- This protocol is written in line with PRISMA guidelines and will include both subgroup and sensitivity analyses to further investigate heterogeneity of included studies.
- AI-specific metrics such as the F1-score and precision-recall AUC will be employed to mitigate the restrictions of conventional pooled analysis, such as the impact of class imbalance.
- As AI is a relatively novel technology in mpMRI, the long-term data on clinical outcomes associated with its use may be limited.
- The restrictions on language and use of multiparametric MRI only may result in few suitable studies for inclusion.

For peer review only

BACKGROUND

Accurately identifying clinically significant prostate cancer has been a long-standing, challenging issue. Previous diagnostic pathways have resulted in many patients undergoing biopsies diagnosed with clinically insignificant prostate cancer. This can lead to potentially aggressive overtreatment, unnecessary healthcare cost and detrimental effects on patient mental health(1-3).

The introduction of multiparametric magnetic resonance imaging (mpMRI) was pivotal in resolving this issue. Multiple sources now recommend using mpMRI as a prebiopsy investigation(2-7) to allow earlier risk stratification of those with suspected prostate cancer and improve the utility of the biopsies performed by enabling a more targeted approach. Despite this, interobserver variability of images remains a major concern(5,8), with a lack of standardised protocols and a requirement for experienced radiologists being examples of contributory factors(4,9). While radiology trainees show rapid

For peer review only

93 improvement in the accuracy of interpreting studies with increased exposure, there is still a margin of error at around 25%(10). Additionally, prostate cancer is the second most frequent cancer diagnosis globally, and the healthcare burden is only set to increase due to a growing ageing population. Therefore, the volume of diagnostic imaging and management monitoring required of radiologists will also likely increase over time(11).

Artificial Intelligence (AI), including Deep Learning (DL), describes how computers can be programmed to imitate human intelligence(12). AI can be utilised in a semiautomated or fully automated approach. Semiautomated AI refers to using more traditional machine learning mechanisms such as radiomics, where radiologists must perform some pre-processing of images to make them algorithm appropriate. Fully automated AI has the benefit of using neural networks to perform feature identification(13). Neural networks are a subset of deep learning models that mimic the human visual cortex(14). Each neural network layer comprises neurons that identify several features of an image by applying multiple edge, colour and texture filters(15). These neural networks are trained to associate specific characteristics with certain classifications by iterating through a training dataset of images. These trained layers can work together to perform feature identification on unseen photos; for example, in mpMRI, they could segment a potentially suspicious lesion in the prostate(16,17). AI is being integrated into clinical practice to support diagnoses, assist in therapeutic decisions, and predict patient outcomes(18). Several studies have assessed the level of agreement between AI and DL to clinical radiologists to interpret mpMRI in prostate cancer investigation(19-22). Application of AI could improve the current

For peer review only

interobserver variability, expedite mpMRI interpretation(21), and risk-stratify potentially suspicious lesions for radiologist review.

However, it is also essential to address that DL algorithms are not yet advanced enough for unsupervised clinical utilisation(19,23). Many issues surround the training of AI models, such as lack of diversity in training datasets – primarily in ethnicity, with many cohorts using predominantly white patients(24), even though prostate cancer is more prevalent and aggressive in Afro-Caribbean men(11). In many studies, there is a lack of external validation, which makes it unclear how models would perform on new data, and also runs the risk of overfitting to training data – where the model is excellent at recognising lesions in training data but is unable to recognise lesions in new data(24). Class imbalance due to most negative cases can also present as an issue by creating algorithm bias and false negatives, which warrants review by senior radiologists(24).

Furthermore, variations of the Prostate Imaging Reporting and Data System (PI-RADS) exist – with version 1 introduced in 2011, replaced by version 2 in 2016, and version 2.1 in 2019(25,26). This makes evaluating how comparable the AI is more challenging due to a lack of consistency. This study will explore this in more detail to inform future reporting methodologies if different PI-RADS versions are used in the included studies. As some previous studies report heterogeneity amongst

For peer review only

studies(13,19,27), we hope to ascertain to the level of heterogeneity in current studies and the impact of specific study methodologies on overall heterogeneity.

This systematic review and meta-analysis aims to summarise the accuracy of AI in predicting prostate cancer on mpMRI and how its use may impact clinical outcomes in patients.

METHODS AND ANALYSIS

The protocol for this systematic review follows the Preferred Reporting Items for Systematic Review and Meta-Analysis Protocols (PRISMA-P) 2015 checklist(28). The study has been prospectively registered with PROSPERO review databases (CRD42021293745), and all methods described here were established before implementation. The statistical data evaluating the sensitivity, specificity, positive predictive value (PPV), negative predictive value (NPV), and area-under-the-curve (AUC) of the use of AI in mpMRI prostate imaging for the detection of prostate cancer will be derived following a thorough analysis and thematic synthesis of included studies. These studies' pooled sensitivities and specificities will be determined before PPV and NPV values are derived.

Search Methodology

A systematic search will be performed using PubMed, MEDLINE, Embase and Cochrane databases. The search strategy will include using medical subject heading (MeSH) terms and free text with appropriate Boolean operators and will encompass articles from January 2016 to February 2023 to

For peer review only

achieve maximum yield of the relevant evidence. The search will include the following key terms: "prostate", "cancer", "diagnosis", and multiple synonyms for "mpMRI", "artificial intelligence", and "deep learning". The full search strategy is detailed in supplementary file 1. To facilitate the initial screening process, Rayyan will be employed, a semiautomated application designed to improve the speed and reporting accuracy of systematic reviews(29). All eligible articles from the initial search will be uploaded to Rayyan. A further manual search of the references in all included articles will be performed to identify any further relevant literature not found by the initial search strategy. If data is absent or ambiguous, the corresponding authors will be contacted for clarification.

Study selection and data extraction

The screening process will be completed independently by two researchers (M.T. and S.M.). Titles and abstracts of eligible studies will be assessed, and irrelevant articles will be removed. A full-text version of relevant articles will be downloaded for further eligibility review. Any dispute amongst researchers will be discussed, and a third reviewer (Y.Z.) will be consulted, with the issue being resolved by consensus. The reasoning for excluding articles will be documented and detailed in a PRISMA flow diagram. Before commencing the screening process, calibration exercises will be conducted to maintain consistency between the researchers, reducing inter-reviewer bias.

Inclusion and exclusion criteria

For peer review only

For inclusion in this systematic review, studies must use either fully automated or semiautomated AI to study multiparametric MRI prostate images to detect prostate cancer. Prospective or retrospective studies can be included. Comparisons will be focussed on evaluating sensitivities, specificities, PPVs, NPVs and AUCs. Patient cohorts can comprise suspected and confirmed incidences of cancer, and studies included should include reference standards using histological findings.

Correspondence papers, ongoing studies, case reports and conference abstracts reports will be excluded from this analysis. Articles which are not written in the English Language will also be excluded. Papers not using multiparametric MRI as the diagnostic modality will be excluded. Studies which include patients with previous treatment of prostate cancer will be excluded.

Data extraction (table of collection)

The following data detailed in Table 1 will be collected from all included studies. The researchers will perform data extraction independently, collating collected data in a dedicated datasheet. Any discrepancies in data extraction will be examined by a third reviewer and will be resolved by consensus.

If the data is available, the relevant figures will be extracted appropriately, including but not limited to true positive, true negative, false positive and false negatives and derivatives of these calculations. If these are not provided, then attempts will be made to calculate from the data provided. If this is unsuccessful, the relevant authors of the paper in question will be contacted to provide the data.

For peer review only

189 **Table 1.** Data collection items

ITEM NO.	DATA TITLE	DATA TYPE
1	Year of publication	Study characteristic
2	Study authors	Study characteristic
3	Experimental design	Study characteristic
4	Patient population	Demographics
5	Study size	Demographics
6	Type of MRI imaging used	Methodology
7	Type of MRI scoring system used	Methodology
8	Type of AI models used	Methodology
9	Definition of clinically significant disease	Methodology
10	Predictive performances	Outcome

190 AI: artificial intelligence; MRI: magnetic resonance imaging

191

192 **Endpoints**

193 The primary endpoint will be statistically significant quantified accuracy in using AI in MRI prostate
194 imaging to detect prostate cancer and determine whether AI could be deployed in the clinical decision-
195 making process. Additional outcomes will include other parameters investigating the extent of the
196 disease.

197

For peer review only

Meta-analysis

A meta-analysis will be conducted if there are a sufficient number of appropriate studies. This would aim to obtain a pooled quantitative diagnostic accuracy value for AI performance detecting prostate cancer from mpMRI. To begin, sensitivity and specificity values will be retrieved, or if studies do not provide these values, they will be calculated from clinical tables or requested from corresponding authors. If a considerable proportion of included studies used another metric, these values would be retrieved and calculated separately. This may include common AI-focused metrics such as F1-score and precision-recall AUC. The distributions of the untransformed, logit and double-arcsine transformed proportions will be compared and assessed for normality using density plots and Shapiro-Wilk tests, with whichever set of ratios most resembling normal distribution being used for further analysis.

Inter-study variation will be quantified through I^{2}, and if statistically significant, a random-effects model will be fitted for estimation of the summary estimate. Once the model fits all relevant studies, leave-one-out analysis (LOO) and accompanying diagnostic plots will be used to identify influential studies. This will include externally studentised residuals, the difference in fits values (DFFITS), Cook's distances, covariance ratios, LOO estimates of the amount of heterogeneity, LOO values of the test statistics for heterogeneity, hat values and weights. Each study will be removed one at a time, and the summary proportions re-estimated based on the remaining n-1 studies. Studies with a statistically significant influence on the fitted model will be considered outliers and removed. The model will then be re-fitted. A summary estimate comprising the remaining studies will then be calculated to estimate the accuracy

For peer review only

using AI in mpMRI for prostate cancer detection. All data analysis and visualisation will be performed using the R statistical environment using the "mvmeta" and "meta" packages.

Risk of bias in individual studies

The QUADAS-2 score will be employed to assess the quality and the risk of bias across the selected studies(30). This tool comprises four domains: patient selection, index test, reference standard, and flow and timing. The quality of the research methodology for each study will be assessed within these four domains. Two (M.T. and Y.Z.) reviewers will be independently involved in this process, with any disparities resolved by consensus. The bias assessment will evaluate the applicability and reliability of the data used. This will inform evidence synthesis and increase transparency as articles may be excluded if found to be of low quality or suggestive of high levels of bias, or if included, appropriate commentaries will be incorporated into the discussion.

Ethics and Dissemination

The potential role of AI in assisting mpMRI interpretation for prostate cancer diagnosis is yet to be evaluated. Through the summation of this information, we hope to clarify the role of AI in improving reporting and summarise its impact on clinical care.

For peer review only

However, several limitations should be highlighted. First, we recognise that due to its relative novelty, there may be a limited number of studies that have evaluated AI in this context. Next, there could be issues surrounding the heterogeneity of reporting protocols due to local variation. Finally, different neural networks may also act as confounders due to different training datasets, which may report differently depending on learned features.

We will publish the results in a peer-reviewed journal. The synthesis of this data will result in a better understanding of current AI and DL protocols. This will hopefully aid in advancing existing systems for supervised and unsupervised usage to optimise reporting protocols in the future and aid prostate cancer diagnosis.

Patient and public involvement

There will be no patient or public involvement in this study.

Trial status:

- Preliminary searches: Started
- Piloting of the study selection process: Started
- Formal screening: Not started
- Data extraction: Not started

For peer review only

- Risk of bias assessment: Not started
- Data analysis: Not started

Author Affiliations

[1] Imperial College London School of Medicine, London, UK

[2] UCL Cancer Institute, University College London, London, UK

[3] Department of Pathology, University College London Hospitals NHS Foundation Trust, London, UK

[4] Department of Radiology, University College London Hospitals NHS Foundation Trust, London, UK

[5] School of Healthcare Sciences, Cardiff University, Wales, UK

[6] UCL Division of Surgery & Interventional Science, University College London, London, UK

[7] Department of Urology, University College London Hospitals NHS Foundation Trust, London, UK

Email: Maya Thomas: maya.thomas18@imperial.ac.uk, Sanjana Murali: sanjana.murali18@imperial.ac.uk, Yi Zhao: yi.zhao18@imperial.ac.uk, Benjamin S. Simpson: b.simpson@ucl.ac.uk, Alex Freeman: alex.freeman2@nhs.net, Alex Kirkam: alexkirkham@nhs.net, Daniel Kelly: kellydm@cardiff.ac.uk, Hayley C. Whitaker: hayley.whitaker@ucl.ac.uk, Mark Emberton: m.emberton@ucl.ac.uk, Joseph M. Norris: joseph.norris@ucl.ac.uk

Twitter Benjamin S. Simpson @b3nsimp, Daniel Kelly @profdkelly, Hayley C. Whitaker @HayleyWhitaker, Mark Emberton @EmbertonMark, Joseph M. Norris @MrJosephNorris

For peer review only

Contributors The authors' contribution includes, but is not limited to, the following: MT and SM wrote the manuscript. YZ and JMN created the study concept. YZ, BSS, AF, AK, DK, HCW and ME provided supervision and guidance. All authors reviewed and approved the manuscript in its current form. MT and SM are the guarantors of this work.

Funding Norris is funded by the Medical Research Council (MRC) (MR/S00680X/1). Simpson is funded by the Royal Marsden Cancer Charity. Emberton receives research support from the United Kingdom's National Institute of Health Research (NIHR) UCLH/UCL Biochemical Research Centre.

Competing Interests Norris receives funding from the MRC. Simpson receives funding from the Royal Marsden Cancer Charity. Whitaker receives funding from Prostate Cancer UK, the Urology Foundation and Rosetrees Trust. Kirkham, Freeman and Emberton have stock interest in Nuada Medical Ltd. Emberton acts as a consultant, trainer and proctor to Sonatherm Inc; Angiodynamics Inc; Exact Imaging Inc.

Patient consent for publication Not required

Provenance and peer review Not commissioned; externally peer-reviewed

Open access This is an open-access article distributed in accordance with the Creative Commons Attribution 4.0 Unreported (CC BY 4.0) license which permits others to copy, redistribute, remix, transform and build upon this work for any purpose, provided the original work is properly cited, a link

For peer review only

to the licence is given, and an indication of whether changes were made. See https://creativecommons.org/licenses/by/4.0/.

ORCID IDs

- Sanjana Murali https://orcid.org/0000-0001-7980-6733
- Maya Thomas https://orcid.org/0000-0001-6997-9438
- Yi Zhao https://orcid.org/0000-0002-4563-4344
- Naomi Morka https://orcid.org/0000-0002-1925-7223
- Joseph M. Norris http://orcid.org/0000-0003-2294-0303
- Benjamin S. Simpson http://orcid.org/0000-0003-3685-6110
- Daniel Kelly http://orcid.org/0000-0002-1847-0655
- Hayley C. Whitaker https://orcid.org/0000-0002-2695-0202
-

References

(1) Sandhu GS, Andriole GL. Overdiagnosis of Prostate Cancer. *Journal of the National Cancer Institute. Monographs.* 2012; 2012 (45): 146-151. 10.1093/jncimonographs/lgs031.

(2) Kasivisvanathan V, Rannikko AS, Borghi M, Panebianco V, Mynderse LA, Vaarala MH, et al. MRI-Targeted or Standard Biopsy for Prostate-Cancer Diagnosis. *New England Journal of Medicine.* 2018; 378 (19): 1767-1777. 10.1056/NEJMoa1801993.

(3) Brown LC, Ahmed HU, Faria R, El-Shater Bosaily A, Gabe R, Kaplan RS, et al. Multiparametric MRI to improve detection of prostate cancer compared with transrectal ultrasound-guided prostate biopsy alone: the PROMIS study. *Health Technology Assessment.* 2018; 22 (39): 1-176. 10.3310/hta22390.

For peer review only

(4) Zhen L, Liu X, Yegang C, Yongjiao Y, Yawei X, Jiaqi K, et al. Accuracy of multiparametric magnetic resonance imaging for diagnosing prostate Cancer: a systematic review and meta-analysis. *BMC cancer.* 2019; 19 (1): 1244. 10.1186/s12885-019-6434-2.

(5) Richenberg J, Logager V, Panebianco V, Rouviere O, Villeirs G, Schoots I. The primacy of multiparametric MRI in men with suspected prostate cancer. *European Radiology.* 2019; 29 (12): 6940-6952. 10.1007/s00330-019-06166-z.

(6) van der Leest M, Cornel E, Israël B, Hendriks R, Padhani AR, Hoogenboom M, et al. Head-to-head Comparison of Transrectal Ultrasound-guided Prostate Biopsy Versus Multiparametric Prostate Resonance Imaging with Subsequent Magnetic Resonance-guided Biopsy in Biopsy-naïve Men with Elevated Prostate-specific Antigen: A Large Prospective Multicenter Clinical Study. *European Urology.* 2019; 75 (4): 570-578. 10.1016/j.eururo.2018.11.023.

(7) Ahmed HU, El-Shater Bosaily A, Brown LC, Gabe R, Kaplan R, Parmar MK, et al. Diagnostic accuracy of multi-parametric MRI and TRUS biopsy in prostate cancer (PROMIS): a paired validating confirmatory study. *Lancet (London, England).* 2017; 389 (10071): 815-822. 10.1016/S0140-6736(16)32401-1.

(8) Stabile A, Giganti F, Rosenkrantz AB, Taneja SS, Villeirs G, Gill IS, et al. Multiparametric MRI for prostate cancer diagnosis: current status and future directions. *Nature Reviews. Urology.* 2020; 17 (1): 41-61. 10.1038/s41585-019-0212-4.

(9) Dominguez C, Plata M, Cataño JG, Palau M, Aguirre D, Narvaez J, et al. Diagnostic accuracy of multiparametric magnetic resonance imaging in detecting extracapsular extension in intermediate and high - risk prostate cancer. *International Brazilian Journal of Urology : official journal of the Brazilian Society of Urology.* 2018; 44 (4): 688-696. 10.1590/S1677-5538.IBJU.2016.0485.

(10) Rosenkrantz AB, Ayoola A, Hoffman D, Khasgiwala A, Prabhu V, Smereka P, et al. The Learning Curve in Prostate MRI Interpretation: Self-Directed Learning Versus Continual Reader Feedback. *AJR. American journal of roentgenology.* 2017; 208 (3): W92-W100. 10.2214/AJR.16.16876.

(11) Rawla P. Epidemiology of Prostate Cancer. *World Journal of Oncology.* 2019; 10 (2): 63-89. 10.14740/wjon1191.

(12) Gore JC. Artificial intelligence in medical imaging. *Magnetic resonance imaging.* 2020; 68 A1-A4. 10.1016/j.mri.2019.12.006.

(13) Sushentsev N, Moreira Da Silva N, Yeung M, Barrett T, Sala E, Roberts M, et al. Comparative performance of fully-automated and semi-automated artificial intelligence methods for the detection of

For peer review only

clinically significant prostate cancer on MRI: a systematic review. *Insights into Imaging.* 2022; 13 (1): 59. 10.1186/s13244-022-01199-3.

(14) van Dyck LE, Kwitt R, Denzler SJ, Gruber WR. Comparing Object Recognition in Humans and Deep Convolutional Neural Networks—An Eye Tracking Study. *Frontiers in Neuroscience.* 2021; 15 750639. 10.3389/fnins.2021.750639.

(15) Saha A, Fadaiefard P, Rabski J, Sadeghian A, Cusimano M. *Machine learning applications using diffusion tensor imaging of human brain: A PubMed literature review.* ; 2020.

(16) Kriegeskorte N, Golan T. Neural network models and deep learning. *Current biology: CB.* 2019; 29 (7): R231-R236. 10.1016/j.cub.2019.02.034.

(17) Schelb P, Kohl S, Radtke JP, Wiesenfarth M, Kickingereder P, Bickelhaupt S, et al. Classification of Cancer at Prostate MRI: Deep Learning versus Clinical PI-RADS Assessment. *Radiology.* 2019; 293 (3): 607-617. 10.1148/radiol.2019190938.

(18) Briganti G, Le Moine O. Artificial Intelligence in Medicine: Today and Tomorrow. *Frontiers in Medicine.* 2020; 7 27. 10.3389/fmed.2020.00027.

(19) Syer T, Mehta P, Antonelli M, Mallett S, Atkinson D, Ourselin S, et al. Artificial Intelligence Compared to Radiologists for the Initial Diagnosis of Prostate Cancer on Magnetic Resonance Imaging: A Systematic Review and Recommendations for Future Studies. *Cancers.* 2021; 13 (13): 3318. 10.3390/cancers13133318.

(20) Van Booven DJ, Kuchakulla M, Pai R, Frech FS, Ramasahayam R, Reddy P, et al. A Systematic Review of Artificial Intelligence in Prostate Cancer. *Research and Reports in Urology.* 2021; 13 31-39. 10.2147/RRU.S268596.

(21) Soerensen SJC, Fan RE, Seetharaman A, Chen L, Shao W, Bhattacharya I, et al. Deep Learning Improves Speed and Accuracy of Prostate Gland Segmentations on Magnetic Resonance Imaging for Targeted Biopsy. *The Journal of Urology.* 2021; 206 (3): 604-612. 10.1097/JU.0000000000001783.

(22) Gentile F, La Civita E, Della Ventura B, Ferro M, Cennamo M, Bruzzese D, et al. A Combinatorial Neural Network Analysis Reveals a Synergistic Behaviour of Multiparametric Magnetic Resonance and Prostate Health Index in the Identification of Clinically Significant Prostate Cancer. *Clinical Genitourinary Cancer.* 2022; 20 (5): e406-e410. 10.1016/j.clgc.2022.04.013.

(23) Li H, Lee CH, Chia D, Lin Z, Huang W, Tan CH. Machine Learning in Prostate MRI for Prostate Cancer: Current Status and Future Opportunities. *Diagnostics (Basel, Switzerland).* 2022; 12 (2): 289. 10.3390/diagnostics12020289.

For peer review only

(24) Bulten W, Kartasalo K, Chen PC, Ström P, Pinckaers H, Nagpal K, et al. Artificial intelligence for diagnosis and Gleason grading of prostate cancer: the PANDA challenge. *Nature Medicine.* 2022; 28 (1): 154-163. 10.1038/s41591-021-01620-2.

(25) Aliukonis P, Letauta T, Briedienė R, Naruševičiūtė I, Letautienė S. The role of different PI-RADS versions in prostate multiparametric magnetic resonance tomography assessment. *Acta Medica Lituanica.* 2017; 24 (1): 44-50. 10.6001/actamedica.v24i1.3462.

(26) Turkbey B, Rosenkrantz AB, Haider MA, Padhani AR, Villeirs G, Macura KJ, et al. Prostate Imaging Reporting and Data System Version 2.1: 2019 Update of Prostate Imaging Reporting and Data System Version 2. *European Urology.* 2019; 76 (3): 340-351. 10.1016/j.eururo.2019.02.033.

(27) Rouvière O, Jaouen T, Baseilhac P, Benomar ML, Escande R, Crouzet S, et al. Artificial intelligence algorithms aimed at characterizing or detecting prostate cancer on MRI: How accurate are they when tested on independent cohorts? - A systematic review. *Diagnostic and Interventional Imaging.* 2023; 104 (5): 221-234. 10.1016/j.diii.2022.11.005.

(28) Moher D, Shamseer L, Clarke M, Ghersi D, Liberati A, Petticrew M, et al. Preferred reporting items for systematic review and meta-analysis protocols (PRISMA-P) 2015 statement. *Systematic Reviews.* 2015; 4 (1): 1. 10.1186/2046-4053-4-1.

(29) Ouzzani M, Hammady H, Fedorowicz Z, Elmagarmid A. Rayyan—a web and mobile app for systematic reviews. *Systematic Reviews.* 2016; 5 10.1186/s13643-016-0384-4.

(30) Whiting PF, Rutjes AWS, Westwood ME, Mallett S, Deeks JJ, Reitsma JB, et al. QUADAS-2: a revised tool for the quality assessment of diagnostic accuracy studies. *Annals of Internal Medicine.* 2011; 155 (8): 529-536. 10.7326/0003-4819-155-8-201110180-00009.

For peer review only

The use of artificial intelligence in the detection of primary prostate cancer in multiparametric magnetic resonance imaging with its clinical outcomes: a protocol for a systematic review and meta-analysis

The search terms for this systematic review are as follows:

((artificial intelligen* OR machine intelligen* OR computational intelligen* OR neural network OR deep learning OR machine learning OR reinforcement learning OR convolutional neural network OR artificial neural network OR recurrent neural network OR deep neural network OR intelligent model OR AI OR NN OR DL OR ML OR CNN OR ANN OR RNN OR DNN) AND ((Prostat* AND (cancer OR malignan* OR adenocarcinoma OR lesion* OR disease)) AND (MR OR magnetic resonance imaging OR MP-MRI OR multi-parametric MRI OR multi-parametric magnetic resonance imaging OR multiparametric MRI OR "multiparametric magnetic resonance imaging" OR radiomic* OR radiogenomic*)) AND diagnosis).ti,ab

Searches will be conducted on the following databases:

PubMed, MEDLINE, Embase, and Cochrane databases

For peer review only

Reporting checklist for protocol of a systematic review and meta analysis.

Based on the PRISMA-P guidelines.

Instructions to authors

Complete this checklist by entering the page numbers from your manuscript where readers will find each of the items listed below.

Your article may not currently address all the items on the checklist. Please modify your text to include the missing information. If you are certain that an item does not apply, please write "n/a" and provide a short explanation.

Upload your completed checklist as an extra file when you submit to a journal.

In your methods section, say that you used the PRISMA-Preporting guidelines, and cite them as:

Moher D, Shamseer L, Clarke M, Ghersi D, Liberati A, Petticrew M, Shekelle P, Stewart LA. Preferred Reporting Items for Systematic Review and Meta-Analysis Protocols (PRISMA-P) 2015 statement. Syst Rev. 2015;4(1):1.

For peer review only

		Reporting Item	Page Number
**Title**			
Identification	#1a	Identify the report as a protocol of a systematic review	1
Update	#1b	If the protocol is for an update of a previous systematic review, identify as such	n/a

Registration

	#2	If registered, provide the name of the registry (such as PROSPERO) and registration number	2

Authors

Contact	#3a	Provide name, institutional affiliation, e-mail address of all protocol authors; provide physical mailing address of corresponding author	1, 10-11
Contribution	#3b	Describe contributions of protocol authors and identify the guarantor of the review	11

Amendments

	#4	If the protocol represents an amendment of a previously completed or published protocol, identify as such and list changes; otherwise, state plan for documenting important protocol amendments	n/a

Support

Sources	#5a	Indicate sources of financial or other support for the review	11
Sponsor	#5b	Provide name for the review funder and / or sponsor	11
Role of sponsor or funder	#5c	Describe roles of funder(s), sponsor(s), and / or institution(s), if any, in developing the protocol	11

Introduction

For peer review only

Rationale	#6	Describe the rationale for the review in the context of what is already known	4
Objectives	#7	Provide an explicit statement of the question(s) the review will address with reference to participants, interventions, comparators, and outcomes (PICO)	5, 6-7
**Methods**			
Eligibility criteria	#8	Specify the study characteristics (such as PICO, study design, setting, time frame) and report characteristics (such as years considered, language, publication status) to be used as criteria for eligibility for the review	6-7
Information sources	#9	Describe all intended information sources (such as electronic databases, contact with study authors, trial registers or other grey literature sources) with planned dates of coverage	6-7
Search strategy	#10	Present draft of search strategy to be used for at least one electronic database, including planned limits, such that it could be repeated	Supplementary file
Study records - data management	#11a	Describe the mechanism(s) that will be used to manage records and data throughout the review	6-7
Study records - selection process	#11b	State the process that will be used for selecting studies (such as two independent reviewers) through	6-7

For peer review only

		each phase of the review (that is, screening, eligibility and inclusion in meta-analysis)	
Study records - data collection process	#11c	Describe planned method of extracting data from reports (such as piloting forms, done independently, in duplicate), any processes for obtaining and confirming data from investigators	7
Data items	#12	List and define all variables for which data will be sought (such as PICO items, funding sources), any pre-planned data assumptions and simplifications	7
Outcomes and prioritization	#13	List and define all outcomes for which data will be sought, including prioritization of main and additional outcomes, with rationale	7-8
Risk of bias in individual studies	#14	Describe anticipated methods for assessing risk of bias of individual studies, including whether this will be done at the outcome or study level, or both; state how this information will be used in data synthesis	9
Data synthesis	#15a	Describe criteria under which study data will be quantitatively synthesised	8
Data synthesis	#15b	If data are appropriate for quantitative synthesis, describe planned summary measures, methods of handling data and methods of combining data from studies, including any planned exploration of consistency (such as I2, Kendall's τ)	8

For peer review only

Data synthesis	#15c	Describe any proposed additional analyses (such as sensitivity or subgroup analyses, meta-regression)	8
Data synthesis	#15d	If quantitative synthesis is not appropriate, describe the type of summary planned	8
Meta-bias(es)	#16	Specify any planned assessment of meta-bias(es) (such as publication bias across studies, selective reporting within studies)	8-9
Confidence in cumulative evidence	#17	Describe how the strength of the body of evidence will be assessed (such as GRADE)	8-9

The PRISMA-P elaboration and explanation paper is distributed under the terms of the Creative Commons Attribution License CC-BY. This checklist was completed on 20. March 2023 using https://www.goodreports.org/, a tool made by the EQUATOR Network in collaboration with Penelope.ai

For peer review only

BMJ Open

The use of artificial intelligence in the detection of primary prostate cancer in multiparametric magnetic resonance imaging with its clinical outcomes: a protocol for a systematic review and meta-analysis

Journal:	*BMJ Open*
Manuscript ID	bmjopen-2023-074009.R3
Article Type:	Protocol
Date Submitted by the Author:	27-Jul-2023
Complete List of Authors:	Thomas, Maya; Imperial College London, School of Medicine<br>Murali, Sanjana; Imperial College London, School of Medicine<br>Simpson, Benjamin Scott; University College London, UCL Cancer Institute<br>Freeman, Alex; University College London Hospitals NHS Foundation Trust, Department of Pathology<br>Kirkham, Alex; University College London Hospitals NHS Foundation Trust, Department of Radiology<br>Kelly, Daniel; Cardiff University, School of Healthcare Sciences<br>Whitaker, Hayley; University College London, UCL Division of Surgery and Interventional Science<br>Zhao, Yi; Imperial College London, School of Medicine<br>Emberton, Mark; University College London, UCL Division of Surgery & Interventional Science; University College London Hospitals NHS Foundation Trust, Department of Urology<br>Norris, Joseph; University College London, UCL Division of Surgery & Interventional Science; University College London Hospitals NHS Foundation Trust, Department of Urology
**Primary Subject Heading**:	Urology
Secondary Subject Heading:	Radiology and imaging, Oncology
Keywords:	Prostate, Urological tumours < UROLOGY, Urological tumours < ONCOLOGY, RADIOLOGY & IMAGING, Magnetic resonance imaging < RADIOLOGY & IMAGING

SCHOLARONE™
Manuscripts

The use of artificial intelligence in the detection of primary prostate cancer in multiparametric magnetic resonance imaging with its clinical outcomes: a protocol for a systematic review and meta-analysis

Maya Thomas[1], Sanjana Murali[1], Benjamin S. Simpson[2], Alex Freeman[3], Alex Kirkham[4], Daniel Kelly[5], Hayley C. Whitaker[6], Yi Zhao[1], Mark Emberton[6,7], Joseph M. Norris[6,7]

MT and SM are joint first authors.

ME and JMN are joint senior authors.

[1] Imperial College London School of Medicine, London, UK

[2] UCL Cancer Institute, University College London, London, UK

[3] Department of Pathology, University College London Hospitals NHS Foundation Trust, London, UK

[4] Department of Radiology, University College London Hospitals NHS Foundation Trust, London, UK

[5] School of Healthcare Sciences, Cardiff University, Wales, UK

[6] UCL Division of Surgery & Interventional Science, University College London, London, UK

[7] Department of Urology, University College London Hospitals NHS Foundation Trust, London, UK

Correspondence to: maya.thomas18@imperial.ac.uk

For peer review only

Imperial College London

Exhibition Rd, South Kensington, London SW7 2BU

maya.thomas18@imperial.ac.uk

Ethics: No ethical considerations apply.

Word count: 1793

For peer review only

ABSTRACT

Introduction: Multiparametric magnetic resonance imaging (mpMRI) has transformed the prostate cancer diagnostic pathway, allowing for improved risk stratification and more targeted subsequent management. However, concerns exist over the interobserver variability of images and the applicability of this model long term, especially considering the current shortage of radiologists and the growing ageing population. Artificial intelligence (AI) is being integrated into clinical practice to support diagnostic and therapeutic imaging analysis to overcome these concerns. The following report details a protocol for a systematic review and meta-analysis investigating the accuracy of AI in predicting primary prostate cancer on mpMRI.

Methods and Analysis: A systematic search will be performed using PubMed, MEDLINE, Embase, and Cochrane databases. All relevant articles published between January 2016 and February 2023 will be eligible for inclusion. To be included, articles must use AI to study MRI prostate images to detect prostate cancer. All included articles will be in full-text, reporting original data, and written in English. The protocol follows the Preferred Reporting Items for Systematic Review and Meta-Analysis Protocol (PRISMA-P) 2015 checklist. The QUADAS-2 score will assess the quality and risk of bias across selected studies.

Ethics and dissemination: Ethical approval will not be required for this systematic review. Findings will be disseminated through peer-reviewed publications and presentations at both national and international conferences.

PROSPERO registration number: CRD42021293745

For peer review only

Strengths and limitations of this study:

- This protocol is written in line with PRISMA guidelines and will include both subgroup and sensitivity analyses to further investigate heterogeneity of included studies.
- AI-specific metrics such as the F1-score and precision-recall AUC will be employed to mitigate the restrictions of conventional pooled analysis, such as the impact of class imbalance.
- As AI is a relatively novel technology in mpMRI, the long-term data on clinical outcomes associated with its use may be limited.
- The restrictions on language and use of multiparametric MRI only may result in few suitable studies for inclusion.

For peer review only

BACKGROUND

Accurately identifying clinically significant prostate cancer has been a long-standing, challenging issue. Previous diagnostic pathways have resulted in many patients undergoing biopsies diagnosed with clinically insignificant prostate cancer. This can lead to potentially aggressive overtreatment, unnecessary healthcare cost and detrimental effects on patient mental health(1-3).

The introduction of multiparametric magnetic resonance imaging (mpMRI) was pivotal in resolving this issue. Multiple sources now recommend using mpMRI as a prebiopsy investigation(2-7) to allow earlier risk stratification of those with suspected prostate cancer and improve the utility of the biopsies performed by enabling a more targeted approach. Despite this, interobserver variability of images remains a major concern(5,8), with a lack of standardised protocols and a requirement for experienced radiologists being examples of contributory factors(4,9). While radiology trainees show rapid

For peer review only

improvement in the accuracy of interpreting studies with increased exposure, there is still a margin of error at around 25%(10). Additionally, prostate cancer is the second most frequent cancer diagnosis globally, and the healthcare burden is only set to increase due to a growing ageing population. Therefore, the volume of diagnostic imaging and management monitoring required of radiologists will also likely increase over time(11).

Artificial Intelligence (AI), including Deep Learning (DL), describes how computers can be programmed to imitate human intelligence(12). AI can be utilised in a semiautomated or fully automated approach. Semiautomated AI refers to using more traditional machine learning mechanisms such as radiomics, where radiologists must perform some pre-processing of images to make them algorithm appropriate. Fully automated AI has the benefit of using neural networks to perform feature identification(13). Neural networks are a subset of deep learning models that mimic the human visual cortex(14). Each neural network layer comprises neurons that identify several features of an image by applying multiple edge, colour and texture filters(15). These neural networks are trained to associate specific characteristics with certain classifications by iterating through a training dataset of images. These trained layers can work together to perform feature identification on unseen photos; for example, in mpMRI, they could segment a potentially suspicious lesion in the prostate(16,17). AI is being integrated into clinical practice to support diagnoses, assist in therapeutic decisions, and predict patient outcomes(18). Several studies have assessed the level of agreement between AI and DL to clinical radiologists to interpret mpMRI in prostate cancer investigation(19-22). Application of AI could improve the current interobserver

For peer review only

variability, expedite mpMRI interpretation(21), and risk-stratify potentially suspicious lesions for radiologist review.

However, it is also essential to address that DL algorithms are not yet advanced enough for unsupervised clinical utilisation(19,23). Many issues surround the training of AI models, such as lack of diversity in training datasets – primarily in ethnicity, with many cohorts using predominantly white patients(24), even though prostate cancer is more prevalent and aggressive in Afro-Caribbean men(11). In many studies, there is a lack of external validation, which makes it unclear how models would perform on new data, and also runs the risk of overfitting to training data – where the model is excellent at recognising lesions in training data but is unable to recognise lesions in new data(24). Class imbalance due to most negative cases can also present as an issue by creating algorithm bias and false negatives, which warrants review by senior radiologists(24).

Furthermore, variations of the Prostate Imaging Reporting and Data System (PI-RADS) exist – with version 1 introduced in 2011, replaced by version 2 in 2016, and version 2.1 in 2019(25,26). This makes evaluating how comparable the AI is more challenging due to a lack of consistency. This study will explore this in more detail to inform future reporting methodologies if different PI-RADS versions are used in the included studies. As some previous studies report heterogeneity amongst studies(13,19,27), we hope to ascertain to the level of heterogeneity in current studies and the impact of specific study methodologies on overall heterogeneity.

For peer review only

This systematic review and meta-analysis aims to summarise the accuracy of AI in predicting prostate cancer on mpMRI and how its use may impact clinical outcomes in patients.

METHODS AND ANALYSIS

The protocol for this systematic review follows the Preferred Reporting Items for Systematic Review and Meta-Analysis Protocols (PRISMA-P) 2015 checklist(28). The study has been prospectively registered with PROSPERO review databases (CRD42021293745), and all methods described here were established before implementation. The statistical data evaluating the sensitivity, specificity, positive predictive value (PPV), negative predictive value (NPV), and area-under-the-curve (AUC) of the use of AI in mpMRI prostate imaging for the detection of prostate cancer will be derived following a thorough analysis and thematic synthesis of included studies. These studies' pooled sensitivities and specificities will be determined before PPV and NPV values are derived.

Search Methodology

A systematic search will be performed using PubMed, MEDLINE, Embase and Cochrane databases. The search strategy will include using medical subject heading (MeSH) terms and free text with appropriate Boolean operators and will encompass articles from January 2016 to February 2023 to achieve maximum yield of the relevant evidence. The search will include the following key terms: "prostate", "cancer", "diagnosis", and multiple synonyms for "mpMRI", "artificial intelligence", and "deep

For peer review only

learning". The full search strategy is detailed in supplementary file 1. To facilitate the initial screening process, Rayyan will be employed, a semiautomated application designed to improve the speed and reporting accuracy of systematic reviews(29). All eligible articles from the initial search will be uploaded to Rayyan. A further manual search of the references in all included articles will be performed to identify any further relevant literature not found by the initial search strategy. If data is absent or ambiguous, the corresponding authors will be contacted for clarification.

Study selection and data extraction

The screening process will be completed independently by two researchers (M.T. and S.M.). Titles and abstracts of eligible studies will be assessed, and irrelevant articles will be removed. A full-text version of relevant articles will be downloaded for further eligibility review. Any dispute amongst researchers will be discussed, and a third reviewer (Y.Z.) will be consulted, with the issue being resolved by consensus. The reasoning for excluding articles will be documented and detailed in a PRISMA flow diagram. Before commencing the screening process, calibration exercises will be conducted to maintain consistency between the researchers, reducing inter-reviewer bias.

Inclusion and exclusion criteria

For inclusion in this systematic review, studies must use either fully automated or semiautomated AI to study multiparametric MRI prostate images to detect prostate cancer. Prospective or retrospective studies can be included. Comparisons will be focussed on evaluating sensitivities, specificities, PPVs,

For peer review only

NPVs and AUCs. Patient cohorts can comprise suspected and confirmed incidences of cancer, and studies included should include reference standards using histological findings.

Correspondence papers, ongoing studies, case reports and conference abstracts reports will be excluded from this analysis. Articles which are not written in the English Language will also be excluded. Papers not using multiparametric MRI as the diagnostic modality will be excluded. Studies which include patients with previous treatment of prostate cancer will be excluded.

Data extraction (table of collection)

The following data detailed in Table 1 will be collected from all included studies. The researchers will perform data extraction independently, collating collected data in a dedicated datasheet. Any discrepancies in data extraction will be examined by a third reviewer and will be resolved by consensus.

If the data is available, the relevant figures will be extracted appropriately, including but not limited to true positive, true negative, false positive and false negatives and derivatives of these calculations. If these are not provided, then attempts will be made to calculate from the data provided. If this is unsuccessful, the relevant authors of the paper in question will be contacted to provide the data.

Table 1. Data collection items

ITEM NO.	DATA TITLE	DATA TYPE
1	Year of publication	Study characteristic

For peer review only

**2**	Study authors	Study characteristic
**3**	Experimental design	Study characteristic
**4**	Patient population	Demographics
**5**	Study size	Demographics
**6**	Type of MRI imaging used	Methodology
**7**	Type of MRI scoring system used	Methodology
**8**	Type of AI models used	Methodology
**9**	Definition of clinically significant disease	Methodology
**10**	Predictive performances	Outcome

AI: artificial intelligence; MRI: magnetic resonance imaging

Endpoints

The primary endpoint will be statistically significant quantified accuracy in using AI in MRI prostate imaging to detect prostate cancer and determine whether AI could be deployed in the clinical decision-making process. Additional outcomes will include other parameters investigating the extent of the disease.

Meta-analysis

A meta-analysis will be conducted if there are a sufficient number of appropriate studies. This would aim to obtain a pooled quantitative diagnostic accuracy value for AI performance detecting prostate

For peer review only

cancer from mpMRI. To begin, sensitivity and specificity values will be retrieved, or if studies do not provide these values, they will be calculated from clinical tables or requested from corresponding authors. If a considerable proportion of included studies used another metric, these values would be retrieved and calculated separately. This may include common AI-focused metrics such as F1-score and precision-recall AUC. The distributions of the untransformed, logit and double-arcsine transformed proportions will be compared and assessed for normality using density plots and Shapiro-Wilk tests, with whichever set of ratios most resembling normal distribution being used for further analysis.

Inter-study variation will be quantified through $I^{2,}$ and if statistically significant, a random-effects model will be fitted for estimation of the summary estimate. Once the model fits all relevant studies, leave-one-out analysis (LOO) and accompanying diagnostic plots will be used to identify influential studies. This will include externally studentised residuals, the difference in fits values (DFFITS), Cook's distances, covariance ratios, LOO estimates of the amount of heterogeneity, LOO values of the test statistics for heterogeneity, hat values and weights. Each study will be removed one at a time, and the summary proportions re-estimated based on the remaining n-1 studies. Studies with a statistically significant influence on the fitted model will be considered outliers and removed. The model will then be re-fitted. A summary estimate comprising the remaining studies will then be calculated to estimate the accuracy using AI in mpMRI for prostate cancer detection. All data analysis and visualisation will be performed using the R statistical environment using the "mvmeta" and "meta" packages.

For peer review only

Risk of bias in individual studies

The QUADAS-2 score will be employed to assess the quality and the risk of bias across the selected studies(30). This tool comprises four domains: patient selection, index test, reference standard, and flow and timing. The quality of the research methodology for each study will be assessed within these four domains. Two (M.T. and Y.Z.) reviewers will be independently involved in this process, with any disparities resolved by consensus. The bias assessment will evaluate the applicability and reliability of the data used. This will inform evidence synthesis and increase transparency as articles may be excluded if found to be of low quality or suggestive of high levels of bias, or if included, appropriate commentaries will be incorporated into the discussion.

Ethics and Dissemination

As primary data is not being collected, no ethical approval is required for this study. The results of this systematic review will be published in a peer-reviewed academic journal and scientific conferences. Any collected data will be made available as supplemental material for full transparency.

Patient and public involvement

There will be no patient or public involvement in this study.

Trial status:

- Preliminary searches: Started

For peer review only

- Piloting of the study selection process: Started
- Formal screening: Not started
- Data extraction: Not started
- Risk of bias assessment: Not started
- Data analysis: Not started

Author Affiliations

[1] Imperial College London School of Medicine, London, UK

[2] UCL Cancer Institute, University College London, London, UK

[3] Department of Pathology, University College London Hospitals NHS Foundation Trust, London, UK

[4] Department of Radiology, University College London Hospitals NHS Foundation Trust, London, UK

[5] School of Healthcare Sciences, Cardiff University, Wales, UK

[6] UCL Division of Surgery & Interventional Science, University College London, London, UK

[7] Department of Urology, University College London Hospitals NHS Foundation Trust, London, UK

Email: Maya Thomas: maya.thomas18@imperial.ac.uk, Sanjana Murali: sanjana.murali18@imperial.ac.uk, Yi Zhao: yi.zhao18@imperial.ac.uk, Benjamin S. Simpson: b.simpson@ucl.ac.uk, Alex Freeman: alex.freeman2@nhs.net, Alex Kirkam: alexkirkham@nhs.net, Daniel Kelly: kellydm@cardiff.ac.uk, Hayley C. Whitaker: hayley.whitaker@ucl.ac.uk, Mark Emberton: m.emberton@ucl.ac.uk, Joseph M. Norris: joseph.norris@ucl.ac.uk

For peer review only

Twitter Benjamin S. Simpson @b3nsimp, Daniel Kelly @profdkelly, Hayley C. Whitaker @HayleyWhitaker, Mark Emberton @EmbertonMark, Joseph M. Norris @MrJosephNorris

Contributors The authors' contribution includes, but is not limited to, the following: MT and SM wrote the manuscript. YZ and JMN created the study concept. YZ, BSS, AF, AK, DK, HCW and ME provided supervision and guidance. All authors reviewed and approved the manuscript in its current form. MT and SM are the guarantors of this work.

Funding Norris is funded by the Medical Research Council (MRC) (MR/S00680X/1). Simpson is funded by the Royal Marsden Cancer Charity. Emberton receives research support from the United Kingdom's National Institute of Health Research (NIHR) UCLH/UCL Biochemical Research Centre.

Competing Interests Norris receives funding from the MRC. Simpson receives funding from the Royal Marsden Cancer Charity. Whitaker receives funding from Prostate Cancer UK, the Urology Foundation and Rosetrees Trust. Kirkham, Freeman and Emberton have stock interest in Nuada Medical Ltd. Emberton acts as a consultant, trainer and proctor to Sonatherm Inc; Angiodynamics Inc; Exact Imaging Inc.

Patient consent for publication Not required

Provenance and peer review Not commissioned; externally peer-reviewed

For peer review only

Open access This is an open-access article distributed in accordance with the Creative Commons Attribution 4.0 Unreported (CC BY 4.0) license which permits others to copy, redistribute, remix, transform and build upon this work for any purpose, provided the original work is properly cited, a link to the licence is given, and an indication of whether changes were made. See https://creativecommons.org/licenses/by/4.0/.

ORCID IDs

- Sanjana Murali https://orcid.org/0000-0001-7980-6733
- Maya Thomas https://orcid.org/0000-0001-6997-9438
- Yi Zhao https://orcid.org/0000-0002-4563-4344
- Naomi Morka https://orcid.org/0000-0002-1925-7223
- Joseph M. Norris http://orcid.org/0000-0003-2294-0303
- Benjamin S. Simpson http://orcid.org/0000-0003-3685-6110
- Daniel Kelly http://orcid.org/0000-0002-1847-0655
- Hayley C. Whitaker https://orcid.org/0000-0002-2695-0202

References

(1) Sandhu GS, Andriole GL. Overdiagnosis of Prostate Cancer. *Journal of the National Cancer Institute. Monographs.* 2012; 2012 (45): 146-151. 10.1093/jncimonographs/lgs031.

(2) Kasivisvanathan V, Rannikko AS, Borghi M, Panebianco V, Mynderse LA, Vaarala MH, et al. MRI-Targeted or Standard Biopsy for Prostate-Cancer Diagnosis. *New England Journal of Medicine.* 2018; 378 (19): 1767-1777. 10.1056/NEJMoa1801993.

For peer review only

(3) Brown LC, Ahmed HU, Faria R, El-Shater Bosaily A, Gabe R, Kaplan RS, et al. Multiparametric MRI to improve detection of prostate cancer compared with transrectal ultrasound-guided prostate biopsy alone: the PROMIS study. *Health Technology Assessment.* 2018; 22 (39): 1-176. 10.3310/hta22390.

(4) Zhen L, Liu X, Yegang C, Yongjiao Y, Yawei X, Jiaqi K, et al. Accuracy of multiparametric magnetic resonance imaging for diagnosing prostate Cancer: a systematic review and meta-analysis. *BMC cancer.* 2019; 19 (1): 1244. 10.1186/s12885-019-6434-2.

(5) Richenberg J, Logager V, Panebianco V, Rouviere O, Villeirs G, Schoots I. The primacy of multiparametric MRI in men with suspected prostate cancer. *European Radiology.* 2019; 29 (12): 6940-6952. 10.1007/s00330-019-06166-z.

(6) van der Leest M, Cornel E, Israël B, Hendriks R, Padhani AR, Hoogenboom M, et al. Head-to-head Comparison of Transrectal Ultrasound-guided Prostate Biopsy Versus Multiparametric Prostate Resonance Imaging with Subsequent Magnetic Resonance-guided Biopsy in Biopsy-naïve Men with Elevated Prostate-specific Antigen: A Large Prospective Multicenter Clinical Study. *European Urology.* 2019; 75 (4): 570-578. 10.1016/j.eururo.2018.11.023.

(7) Ahmed HU, El-Shater Bosaily A, Brown LC, Gabe R, Kaplan R, Parmar MK, et al. Diagnostic accuracy of multi-parametric MRI and TRUS biopsy in prostate cancer (PROMIS): a paired validating confirmatory study. *Lancet (London, England).* 2017; 389 (10071): 815-822. 10.1016/S0140-6736(16)32401-1.

(8) Stabile A, Giganti F, Rosenkrantz AB, Taneja SS, Villeirs G, Gill IS, et al. Multiparametric MRI for prostate cancer diagnosis: current status and future directions. *Nature Reviews. Urology.* 2020; 17 (1): 41-61. 10.1038/s41585-019-0212-4.

(9) Dominguez C, Plata M, Cataño JG, Palau M, Aguirre D, Narvaez J, et al. Diagnostic accuracy of multiparametric magnetic resonance imaging in detecting extracapsular extension in intermediate and high - risk prostate cancer. *International Brazilian Journal of Urology : official journal of the Brazilian Society of Urology.* 2018; 44 (4): 688-696. 10.1590/S1677-5538.IBJU.2016.0485.

(10) Rosenkrantz AB, Ayoola A, Hoffman D, Khasgiwala A, Prabhu V, Smereka P, et al. The Learning Curve in Prostate MRI Interpretation: Self-Directed Learning Versus Continual Reader Feedback. *AJR. American journal of roentgenology.* 2017; 208 (3): W92-W100. 10.2214/AJR.16.16876.

(11) Rawla P. Epidemiology of Prostate Cancer. *World Journal of Oncology.* 2019; 10 (2): 63-89. 10.14740/wjon1191.

For peer review only

(12) Gore JC. Artificial intelligence in medical imaging. *Magnetic resonance imaging.* 2020; 68 A1-A4. 10.1016/j.mri.2019.12.006.

(13) Sushentsev N, Moreira Da Silva N, Yeung M, Barrett T, Sala E, Roberts M, et al. Comparative performance of fully-automated and semi-automated artificial intelligence methods for the detection of clinically significant prostate cancer on MRI: a systematic review. *Insights into Imaging.* 2022; 13 (1): 59. 10.1186/s13244-022-01199-3.

(14) van Dyck LE, Kwitt R, Denzler SJ, Gruber WR. Comparing Object Recognition in Humans and Deep Convolutional Neural Networks—An Eye Tracking Study. *Frontiers in Neuroscience.* 2021; 15 750639. 10.3389/fnins.2021.750639.

(15) Saha A, Fadaiefard P, Rabski J, Sadeghian A, Cusimano M. *Machine learning applications using diffusion tensor imaging of human brain: A PubMed literature review.* ; 2020.

(16) Kriegeskorte N, Golan T. Neural network models and deep learning. *Current biology: CB.* 2019; 29 (7): R231-R236. 10.1016/j.cub.2019.02.034.

(17) Schelb P, Kohl S, Radtke JP, Wiesenfarth M, Kickingereder P, Bickelhaupt S, et al. Classification of Cancer at Prostate MRI: Deep Learning versus Clinical PI-RADS Assessment. *Radiology.* 2019; 293 (3): 607-617. 10.1148/radiol.2019190938.

(18) Briganti G, Le Moine O. Artificial Intelligence in Medicine: Today and Tomorrow. *Frontiers in Medicine.* 2020; 7 27. 10.3389/fmed.2020.00027.

(19) Syer T, Mehta P, Antonelli M, Mallett S, Atkinson D, Ourselin S, et al. Artificial Intelligence Compared to Radiologists for the Initial Diagnosis of Prostate Cancer on Magnetic Resonance Imaging: A Systematic Review and Recommendations for Future Studies. *Cancers.* 2021; 13 (13): 3318. 10.3390/cancers13133318.

(20) Van Booven DJ, Kuchakulla M, Pai R, Frech FS, Ramasahayam R, Reddy P, et al. A Systematic Review of Artificial Intelligence in Prostate Cancer. *Research and Reports in Urology.* 2021; 13 31-39. 10.2147/RRU.S268596.

(21) Soerensen SJC, Fan RE, Seetharaman A, Chen L, Shao W, Bhattacharya I, et al. Deep Learning Improves Speed and Accuracy of Prostate Gland Segmentations on Magnetic Resonance Imaging for Targeted Biopsy. *The Journal of Urology.* 2021; 206 (3): 604-612. 10.1097/JU.0000000000001783.

(22) Gentile F, La Civita E, Della Ventura B, Ferro M, Cennamo M, Bruzzese D, et al. A Combinatorial Neural Network Analysis Reveals a Synergistic Behaviour of Multiparametric Magnetic Resonance and Prostate Health Index in the Identification of Clinically Significant Prostate Cancer. *Clinical Genitourinary Cancer.* 2022; 20 (5): e406-e410. 10.1016/j.clgc.2022.04.013.

For peer review only

(23) Li H, Lee CH, Chia D, Lin Z, Huang W, Tan CH. Machine Learning in Prostate MRI for Prostate Cancer: Current Status and Future Opportunities. *Diagnostics (Basel, Switzerland).* 2022; 12 (2): 289. 10.3390/diagnostics12020289.

(24) Bulten W, Kartasalo K, Chen PC, Ström P, Pinckaers H, Nagpal K, et al. Artificial intelligence for diagnosis and Gleason grading of prostate cancer: the PANDA challenge. *Nature Medicine.* 2022; 28 (1): 154-163. 10.1038/s41591-021-01620-2.

(25) Aliukonis P, Letauta T, Briedienė R, Naruševičiūtė I, Letautienė S. The role of different PI-RADS versions in prostate multiparametric magnetic resonance tomography assessment. *Acta Medica Lituanica.* 2017; 24 (1): 44-50. 10.6001/actamedica.v24i1.3462.

(26) Turkbey B, Rosenkrantz AB, Haider MA, Padhani AR, Villeirs G, Macura KJ, et al. Prostate Imaging Reporting and Data System Version 2.1: 2019 Update of Prostate Imaging Reporting and Data System Version 2. *European Urology.* 2019; 76 (3): 340-351. 10.1016/j.eururo.2019.02.033.

(27) Rouvière O, Jaouen T, Baseilhac P, Benomar ML, Escande R, Crouzet S, et al. Artificial intelligence algorithms aimed at characterizing or detecting prostate cancer on MRI: How accurate are they when tested on independent cohorts? - A systematic review. *Diagnostic and Interventional Imaging.* 2023; 104 (5): 221-234. 10.1016/j.diii.2022.11.005.

(28) Moher D, Shamseer L, Clarke M, Ghersi D, Liberati A, Petticrew M, et al. Preferred reporting items for systematic review and meta-analysis protocols (PRISMA-P) 2015 statement. *Systematic Reviews.* 2015; 4 (1): 1. 10.1186/2046-4053-4-1.

(29) Ouzzani M, Hammady H, Fedorowicz Z, Elmagarmid A. Rayyan—a web and mobile app for systematic reviews. *Systematic Reviews.* 2016; 5 10.1186/s13643-016-0384-4.

(30) Whiting PF, Rutjes AWS, Westwood ME, Mallett S, Deeks JJ, Reitsma JB, et al. QUADAS-2: a revised tool for the quality assessment of diagnostic accuracy studies. *Annals of Internal Medicine.* 2011; 155 (8): 529-536. 10.7326/0003-4819-155-8-201110180-00009.

For peer review only

The use of artificial intelligence in the detection of primary prostate cancer in multiparametric magnetic resonance imaging with its clinical outcomes: a protocol for a systematic review and meta-analysis

The search terms for this systematic review are as follows:

((artificial intelligen* OR machine intelligen* OR computational intelligen* OR neural network OR deep learning OR machine learning OR reinforcement learning OR convolutional neural network OR artificial neural network OR recurrent neural network OR deep neural network OR intelligent model OR AI OR NN OR DL OR ML OR CNN OR ANN OR RNN OR DNN) AND ((Prostat* AND (cancer OR malignan* OR adenocarcinoma OR lesion* OR disease)) AND (MR OR magnetic resonance imaging OR MP-MRI OR multi-parametric MRI OR multi-parametric magnetic resonance imaging OR multiparametric MRI OR “multiparametric magnetic resonance imaging” OR radiomic* OR radiogenomic*)) AND diagnosis).ti,ab

Searches will be conducted on the following databases:

PubMed, MEDLINE, Embase, and Cochrane databases

For peer review only

Reporting checklist for protocol of a systematic review and meta analysis.

Based on the PRISMA-P guidelines.

Instructions to authors

Complete this checklist by entering the page numbers from your manuscript where readers will find each of the items listed below.

Your article may not currently address all the items on the checklist. Please modify your text to include the missing information. If you are certain that an item does not apply, please write "n/a" and provide a short explanation.

Upload your completed checklist as an extra file when you submit to a journal.

In your methods section, say that you used the PRISMA-Preporting guidelines, and cite them as:

Moher D, Shamseer L, Clarke M, Ghersi D, Liberati A, Petticrew M, Shekelle P, Stewart LA. Preferred Reporting Items for Systematic Review and Meta-Analysis Protocols (PRISMA-P) 2015 statement. Syst Rev. 2015;4(1):1.

		Reporting Item	Page Number
**Title**			
Identification	#1a	Identify the report as a protocol of a systematic review	1
Update	#1b	If the protocol is for an update of a previous systematic review, identify as such	n/a

For peer review only

**Registration**			
	#2	If registered, provide the name of the registry (such as PROSPERO) and registration number	2
**Authors**			
Contact	#3a	Provide name, institutional affiliation, e-mail address of all protocol authors; provide physical mailing address of corresponding author	1, 10-11
Contribution	#3b	Describe contributions of protocol authors and identify the guarantor of the review	11
**Amendments**			
	#4	If the protocol represents an amendment of a previously completed or published protocol, identify as such and list changes; otherwise, state plan for documenting important protocol amendments	n/a
**Support**			
Sources	#5a	Indicate sources of financial or other support for the review	11
Sponsor	#5b	Provide name for the review funder and / or sponsor	11
Role of sponsor or funder	#5c	Describe roles of funder(s), sponsor(s), and / or institution(s), if any, in developing the protocol	11
**Introduction**			

For peer review only

Rationale	#6	Describe the rationale for the review in the context of what is already known	4
Objectives	#7	Provide an explicit statement of the question(s) the review will address with reference to participants, interventions, comparators, and outcomes (PICO)	5, 6-7
**Methods**			
Eligibility criteria	#8	Specify the study characteristics (such as PICO, study design, setting, time frame) and report characteristics (such as years considered, language, publication status) to be used as criteria for eligibility for the review	6-7
Information sources	#9	Describe all intended information sources (such as electronic databases, contact with study authors, trial registers or other grey literature sources) with planned dates of coverage	6-7
Search strategy	#10	Present draft of search strategy to be used for at least one electronic database, including planned limits, such that it could be repeated	Supplementary file
Study records - data management	#11a	Describe the mechanism(s) that will be used to manage records and data throughout the review	6-7
Study records - selection process	#11b	State the process that will be used for selecting studies (such as two independent reviewers) through	6-7

For peer review only

		each phase of the review (that is, screening, eligibility and inclusion in meta-analysis)	
Study records - data collection process	#11c	Describe planned method of extracting data from reports (such as piloting forms, done independently, in duplicate), any processes for obtaining and confirming data from investigators	7
Data items	#12	List and define all variables for which data will be sought (such as PICO items, funding sources), any pre-planned data assumptions and simplifications	7
Outcomes and prioritization	#13	List and define all outcomes for which data will be sought, including prioritization of main and additional outcomes, with rationale	7-8
Risk of bias in individual studies	#14	Describe anticipated methods for assessing risk of bias of individual studies, including whether this will be done at the outcome or study level, or both; state how this information will be used in data synthesis	9
Data synthesis	#15a	Describe criteria under which study data will be quantitatively synthesised	8
Data synthesis	#15b	If data are appropriate for quantitative synthesis, describe planned summary measures, methods of handling data and methods of combining data from studies, including any planned exploration of consistency (such as I2, Kendall's τ)	8

For peer review only

Data synthesis	#15c	Describe any proposed additional analyses (such as sensitivity or subgroup analyses, meta-regression)	8
Data synthesis	#15d	If quantitative synthesis is not appropriate, describe the type of summary planned	8
Meta-bias(es)	#16	Specify any planned assessment of meta-bias(es) (such as publication bias across studies, selective reporting within studies)	8-9
Confidence in cumulative evidence	#17	Describe how the strength of the body of evidence will be assessed (such as GRADE)	8-9

The PRISMA-P elaboration and explanation paper is distributed under the terms of the Creative Commons Attribution License CC-BY. This checklist was completed on 20. March 2023 using https://www.goodreports.org/, a tool made by the EQUATOR Network in collaboration with Penelope.ai

For peer review only
